# Connectivity differs by orders of magnitude among co-distributed corals, affecting spatial scales of eco-evolutionary processes

**DOI:** 10.1126/sciadv.adt2066

**Published:** 2025-07-02

**Authors:** Zoe Meziere, Katharine Prata, Marine Lechene, Renata Ferrari, Iva Popovic, Cynthia Riginos

**Affiliations:** ^1^ School of the Environment, The University of Queensland, St Lucia, QLD, Australia.; ^2^ Australian Institute of Marine Science, Townsville, QLD, Australia.; ^3^ James Cook University, Townsville, QLD, Australia.

## Abstract

Reef building corals are declining worldwide, yet the processes driving population connectivity remain poorly understood. Using complementary analyses, we provide quantitative estimates of ecologically relevant dispersal and evolutionarily important gene flow in co-distributed coral species on the Great Barrier Reef. We find dispersal distances across meters (23 to 102 meters) in the brooding *Stylophora pistillata* and across kilometers (21 to 52 kilometers) in the broadcast spawning *Pocillopora verrucosa*, consistent with expectations based on their reproductive modes. Similarly, while gene flow rates averaged over the past ~400,000 generations are very low among *S. pistillata* populations, *P. verrucosa* populations are well connected. As a result, estimates of genetic diversity across multiple metrics and population sizes are smaller in *S. pistillata* compared to *P. verrucosa*. These results highlight the importance of spatial scales and reproductive modes in predicting coral adaptive responses and underscore the value of considering spatial connections between target populations in conservation and restoration.

## Teaser

Dispersal and gene flow differ among corals of the Great Barrier Reef, affecting genetic diversity and adaptive responses.

## INTRODUCTION

Coral reefs are some of the most biodiverse ecosystems worldwide yet are also among the most imperiled facing climate change (*1*). Scleractinian corals are the key ecosystem builders but are immediately threatened by acute disturbances, such as increasingly frequent and severe marine heat waves and storms, and by the compounding effects of chronic stressors, such as increasing average temperatures and ocean acidification (*2*). To persist despite rapidly changing environmental conditions, coral populations must shift their geographical distributions to match local conditions and/or adapt in situ to the changed conditions (*3*). For both types of responses, connectivity across short and long distances is a critical process, determining the movement of coral larvae and the distribution of genetic diversity across the seascape.

Connectivity across a species’ range depends on the movement of propagules or dispersive individuals (*4*, *5*). A species’ dispersal potential is characterized by the distance (i.e., how far individuals or propagules disperse) and the propensity (i.e., how often they disperse). At local scales, the mean dispersal distance determines the spatial extent of the genetic neighborhood, within which most dispersal events occur (*6*). Frequent dispersal greatly affects contemporary ecological processes [within the past 10 to 20 generations, following (*7*)] such as the maintenance of census population sizes (*8*) or population replenishment after disturbance. Rarer long-distance dispersal can facilitate range expansion and is critical for evolutionary processes [more than 10 to 20 generations ago, following (*7*)] as it increases gene flow across larger spatial scales. Gene flow across an extended geographical range influences population genetic differentiation and levels of genetic diversity within populations by counteracting the effects of genetic drift (*9*, *10*). Thus, estimates of generational dispersal distance and gene flow rates across long distances provide valuable insights into ecological and evolutionary dynamics.

Both short- and long-distance dispersal influence the spatial extent at which natural selection operates and therefore affects a species’ adaptive potential. At the scale of the genetic neighborhood, adaptation to local conditions requires very strong selection to overcome genetic homogenization caused by frequent dispersal (*11*, *12*). Across a species’ range, gene flow can enable adaptation by either increasing standing genetic diversity or directly facilitating the spread of beneficial alleles (*13*). Thus, considering the full breath of a species’ connectivity dynamics at varying spatial extents (from a few meters to hundreds of kilometers) is critical to understanding its adaptive capacity.

Complementary genomic methods can offer insights into the genetic consequences of realized dispersal over both short and long distances. For continuously distributed populations, isolation-by-distance (IbD) theory is particularly useful to examine patterns of frequent and contemporary dispersal events. Under an IbD model, genetic dissimilarity between individuals continually increases with increasing geographic distance (*6*, *14*). Combined with information on population size or density, it is possible to estimate a per-generation axial dispersal distance ( σ ) and thereby also estimate the spatial extent of the local genetic neighborhood (*15*, *16*). However, to investigate long-distance dispersal events that have more influence at evolutionary timescales, genomic tools that can detect signals of gene flow between distant populations that rarely exchange genes are most appropriate (*17*). Demographic modeling [e.g., (*18*–*20*)] is particularly relevant as these models can decouple the effects of genetic drift, measured as effective population size (*N_e_*), and migration rates (*m*, the proportion of migrant individuals) on gene flow (*N_e_m*, the number of migrants individuals) (*17*). In addition, demographic models can accommodate asymmetrical gene flow, which is useful for marine species as oceanic currents likely drive directional gene flow over long distances (*21*).

Although fundamental theories and tools to characterize dispersal and gene flow at different spatial extents are available, no studies to date—terrestrial or marine—have used them in tandem. For corals, demographic modeling has provided estimates of long-term gene flow between coral populations for several species [e.g., (*22*–*24*)]. To date, however, only four studies have rigorously quantified dispersal distances using IbD methods (*25*–*28*).

Corals have two distinct reproductive modes. Broadcast spawning species have external gamete fertilization that results in small larvae that settle on the benthos after days to weeks of being pelagic. In contrast, brooding species have internal fertilization, resulting in larger larvae that are often competent to settle on the benthos within hours [reviewed in (*29*)]. Although other factors also affect realized dispersal (e.g., physical environment, larval behavior, and asexual reproduction as polyp bail out or colony fragmentation), we generally expect more extensive connectivity in spawning species compared to brooding species. Previous studies have revealed that population genetic structure can differ among co-distributed coral species and is often correlated with the reproductive mode [e.g., (*30*–*34*)]. However, metrics of population differentiation do not directly quantify gene flow [due to nonrealistic model assumptions (*35*, *36*)] or relative dispersal capacities. In addition, patterns of population structure can be skewed by the presence of undetected cryptic species, which are common in corals (*37*). Resolving cryptic species and coupling complementary analyses that capture dispersal and gene flow over short and long distances could yield powerful inferences of coral connectivity dynamics.

Given accelerating declines of coral populations globally, knowledge about coral species’ dispersal capacities is urgently needed to guide adequate spatial extents for conservation, monitoring, and management. Knowledge regarding dispersal frequency and distances can, for example, be used to decide on the size and spacing of marine protected areas (*29*, *38*, *39*). In addition, with an estimated increase of 1° to 2°C above preindustrial temperatures in the next 30 to 80 years (*40*), coral restoration actions based on manipulating spatial connections are rapidly gaining interest (*41*, *42*). For instance, the translocation of corals colonies or gametes outside of the species’ current range (i.e., assisted migration) or between populations within the species’ current range (i.e., assisted gene flow) are increasingly being considered (*43*) and implemented (*44*). Quantifying genetic diversity, dispersal distances, and gene flow rates can provide expectations for the adaptive capacity and future persistence of populations within a species’ range (*45*) and can also identify suitable source and destination populations for translocation (*37*). Obtaining these estimates for representative coral taxa with different reproductive modes will start to inform the range of spatial scales for eco-evolutionary processes among coral species, which in turn, could guide conservation and restoration practices at the multi-species level.

In this study, we characterize ecologically relevant dispersal and evolutionarily significant gene flow to investigate the multiple spatial extents of connectivity in two common, closely related and co-occurring coral species of the Pocilloporidae family: the brooding species *Stylophora pistillata*, and the spawning species *Pocillopora verrucosa*. This study was conducted along the expanse of the Great Barrier Reef (GBR), Australia, to study processes acting at small (few meters) and large (up to 2200 km) spatial extents. By sampling corals at sites separated by varying geographical distances, using genome-wide sequencing and a comparative framework, we aim to: (i) measure generational dispersal distances, (ii) quantify long-term gene flow between populations, and (iii) investigate their combined impacts on the spatial partitioning on genetic diversity and population differentiation. By considering two dominant coral species with contrasting reproductive modes, our results indicate the range of possible dispersal outcomes across coral species and thereby provide connectivity-based guidelines for coral conservation and restoration.

## RESULTS

### Filtering of genomic data and clonality

**Fig. 1.**
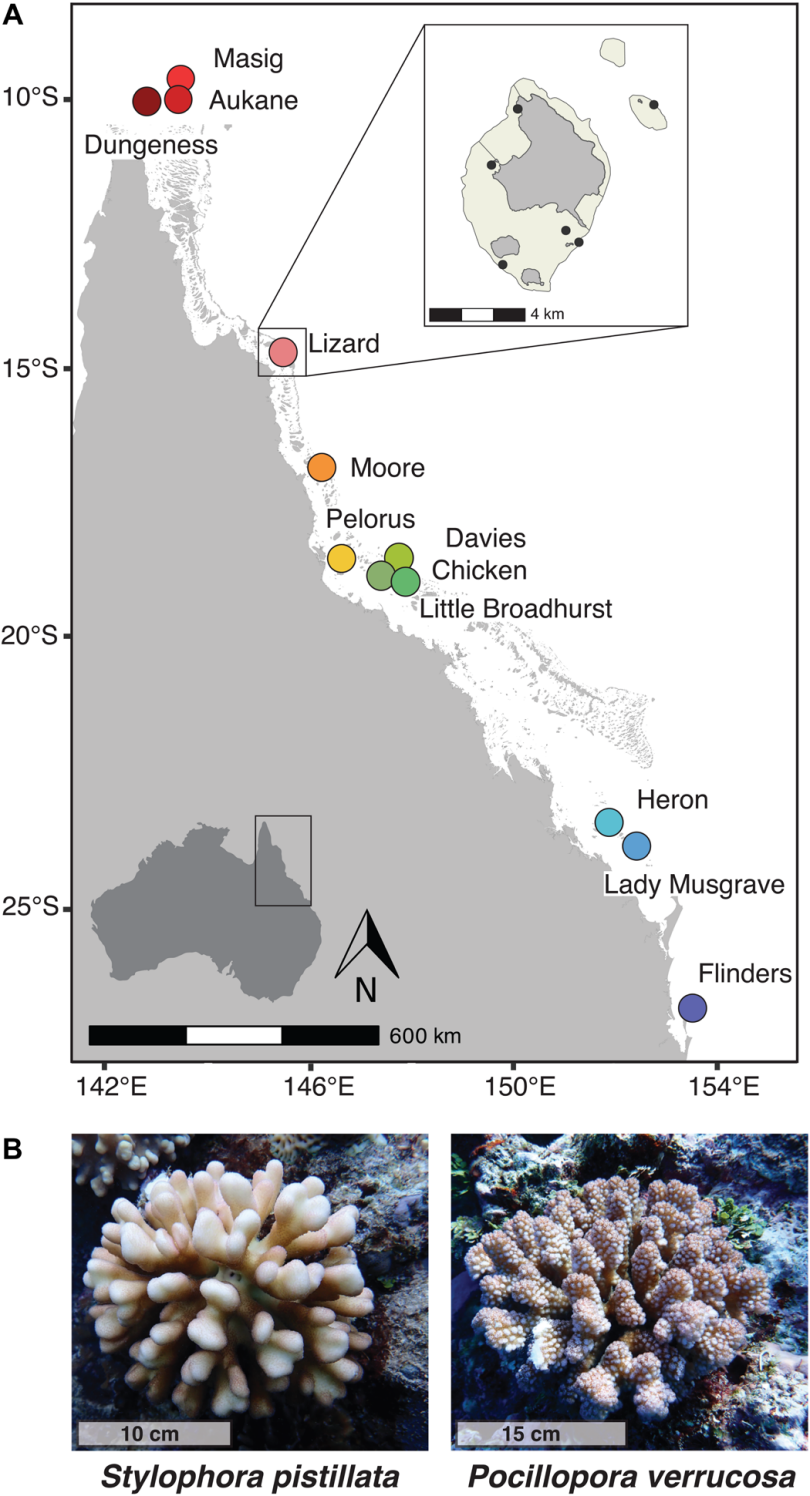
Sampling locations of *Stylophora pistillata* and *Pocillopora verrucosa* coral colonies across the GBR, Australia. (**A**) Map showing the 12 reefs where coral colonies were sampled. Inset shows the six sampling sites at Lizard Island (black dots) as an example to understand the nested sampling design. Maps showing the sampling sites at the other reefs can be found in the Supplementary Materials (fig. S10). (**B**) Photographs of an *S. pistillata* colony (left) and an *S. verrucosa* colony (right). Photo credit: Z. Meziere, the University of Queensland.

Coral colonies were sampled across the GBR, Eastern Australia, from 8 reefs for the brooding species *S. pistillata* and from 12 reefs for the broadcast spawning species *P. verrucosa* (Fig. 1 and tables S1 and S2). Preliminary analyses revealed multiple cryptic taxa [defined as distinct genetic groups that are sympatric, following (*37*)] in both *S. pistillata* (*46*) and *P. verrucosa*, decreasing sample sizes from total number of genotyped colonies. In this study, we only included samples belonging to one cryptic taxon for each nominal species (see Materials and Methods). Reduced representation [double-digest restriction-site associated DNA (ddRAD)] sequencing resulted in an average of 1.3 million raw reads per sample for *S. pistillata* and 3.9 million raw reads per sample for *P. verrucosa*. After alignment to a reference genome and initial IPYRAD filtering, we recovered 10,400 RAD loci for *S. pistillata* and 17,409 RAD loci for *P. verrucosa*. For population structure assessment, isolation by distance analyses and demographic modeling, we performed additional filtering steps and retained 232 samples and 4527 single-nucleotide polymorphisms (SNPs) with less than 5% missing data for *S. pistillata*, and 224 samples and 8597 SNPs with less than 20% missing data for *P. verrucosa* (missingness filtering thresholds and effects are further discussed in the Supplementary Materials). For genetic diversity estimation, we generated all-sites (including non-variant sites) VCF files, which retained 1,265,439 sites (5% missing data) and 207,530 sites (no missing data) for *S. pistillata* and 4,890,746 sites (20% missing data) and 127,977 sites (no missing data) for *P. verrucosa*. We note that using a 99% allelic similarity threshold, we found nine pairs of clonal colonies in *S. pistillata* and two in *P. verrucosa* and removed one representative of each pair from all datasets.

### Reef-level population structure in the brooder *S. pistillata* but lack of population structure in the spawner *P. verrucosa*

**Fig. 2.**
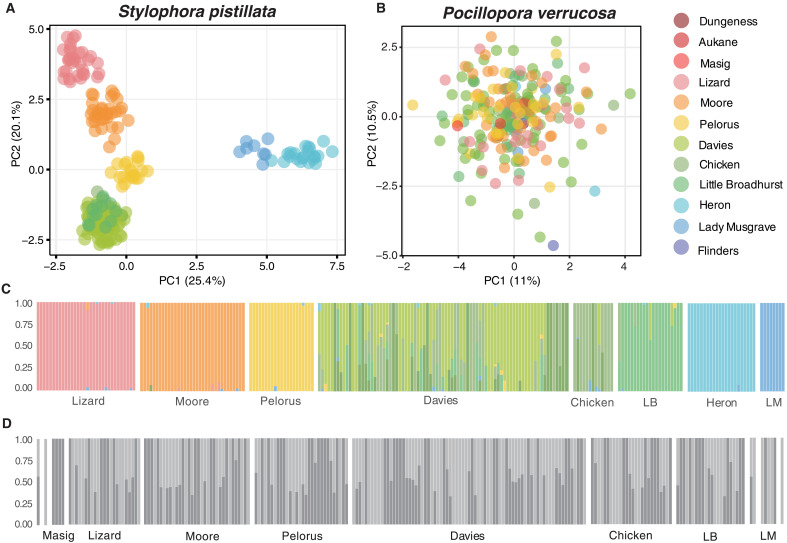
Contrasting patterns of genetic variation partitioning between *S. pistillata* and *P. verrucosa* across the GBR. Reef-level population structure in *S. pistillata* is visualized using the first and second axis of a PCA (**A**) and ancestry proportions obtained in AMIXTURE with *K* = 9 (**C**). Panmixia in *P. verrucosa* is visualized using the first and second axis of a PCA (**B**) and ancestry proportions obtained in AMIXTURE with *K* = 2 (**D**). Reefs are color coded to match Fig. 1. LM, Lady Musgrave; LB, Little Broadhurst.

Investigations of population structure, using principal components analysis (PCA) and ADMIXTURE (*47*), revealed contrasting patterns between species across the sampling range. For *S. pistillata*, PCAs largely reflected the geographic distribution of the reefs sampled and clustered samples into six distinct genetic groups (Fig. 2A and fig. S1). The first two PC axes captured 45.4% of the total genetic variation between samples retained by the first 10 PC axes. Along PC1, samples from the southernmost reefs (Heron and Lady Musgrave) formed two adjacent genetic groups and were separated from the rest of the samples. Along PC2, samples from Lizard, Moore, and Orpheus, formed three distinct groups and samples from the offshore central reefs (Davies, Little Broadhurst. and Chicken) formed another cohesive group. In agreement with the PCA, ADMIXTURE revealed the same reef-level clustering and little admixture among populations, with few samples showing mixed ancestry proportions (i.e., *Q* < 0.80) (Fig. 2C). *K* = 9 was the optimal *K* according to the cross-validation errors and log likelihoods (fig. S3). Pairwise F_ST_ between populations varied from 0.011 (Little Broadhurst–Davies) to 0.146 (Lizard–Heron) (fig. S4). For *P. verrucosa*, the PCA revealed no population structure (Fig. 2B and fig. S2), which was consistent at different levels of missing data (fig. S12). The ADMIXTURE analysis supported *K* = 2 as the best number of groups (Fig. 2D and fig. S3), with no consistent differences in ancestry patterns among reefs. Pairwise F_ST_ between populations varied from 0 to 0.048 (fig. S4), with only small differences at different levels of missing data (fig. S13).

### Generational dispersal across meters in the brooder *S. pistillata* and across kilometers in the spawner *P. verrucosa*

**Fig. 3.**
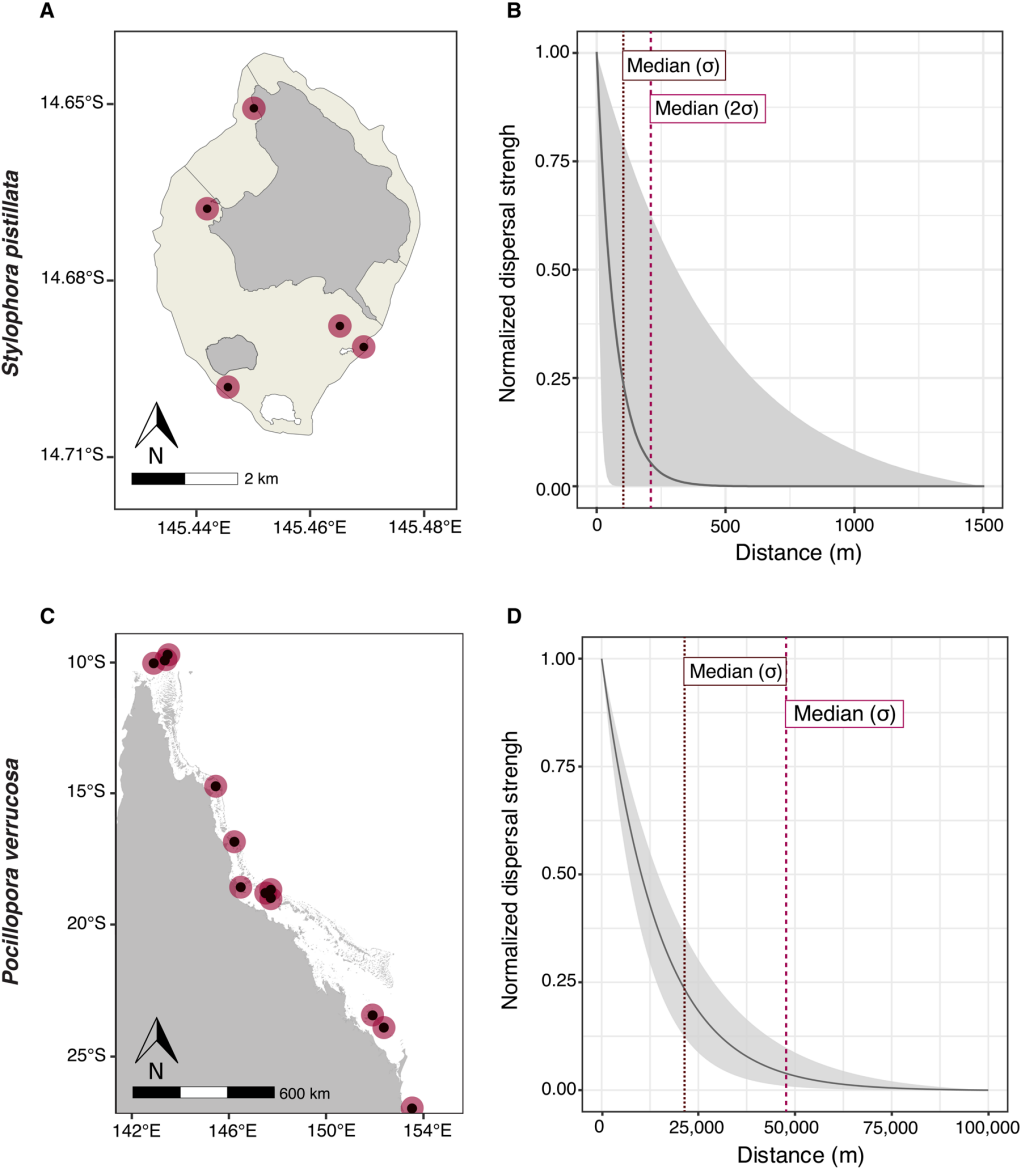
The spatial extent of dispersal varies by order of magnitudes between the brooding coral *S. pistillata* and the spawning coral *P. verrucosa*. (**A**) Median dispersal neighborhood areas for *S. pistillata* (radius of 2 σ = 204 m, red circles), mapped around the sampling sites (black dots) at Lizard island (island in gray, reef area in beige); (**B**) Laplacian dispersal kernel for *S. pistillata*, calibrated using median effective σ (dark gray line) and the 90% probability interval around median σ (light gray shade); (**C**) median dispersal neighborhoods areas for *P. verrucosa* (radius of 2 σ = 42,000 m, red circles), mapped around the sampled reefs (black dotes) on the GBR; (**D**) Laplacian dispersal kernel for *P. verrucosa*, calibrated using median effective σ (dark gray line) and the 90% probability interval around median σ (light gray shade). Effective σ is the axial dispersal distance (i.e., dispersal spread) inferred from an isolation by distance regression slope and an effective population density estimate. Under a Laplace distribution, the dispersal distance is equal to $\frac{\sigma }{\sqrt{2}}$.

Isolation by distance (IbD) regressions performed in the “genepop” R package (*48*) were significant for both species. For *S. pistillata*, we calculated genetic and geographic distances between colonies at the reef level (0 to 10,000 m), to account for the strong signals of population structure, and thus used a two-dimensional (2D) IbD model (see Materials and Methods). With a median regression slope of 0.0032 [95% confidence interval (CI): 0.0021 to 0.0041] (fig. S5), we estimated a median neighborhood size *NS* of 318 colonies [interquartile range (IQR): 286 to 356], representing the number of adults in a random-mating area. Using census counts (table S6) scaled by the abundance of *S. pistillata* Taxon1 compared to other cryptic taxa at each site (see Materials and Methods), we estimated a median census density (*D*_c_) of 0.046 colonies /m^2^ (IQR: 0.045 to 0.048). Using contemporary *N_e_* estimates for each population (Table 1) and surface reef area (table S5), we estimated a median effective density (*D*_e_) of 0.0025 colonies /m^2^ (IQR: 0.0015 to 0.0033). Using these density estimates, we calculated a median axial dispersal distance σ (i.e., dispersal spread) equal to 23 m using *D*_c_ (IQR: 22 to 25) and 102 m using *D*_e_ (IQR: 85 to 140) (Table 1). The probability distribution of effective σ was bimodal, with the major mode at 98 m and the secondary mode at 655 m. The secondary mode was driven by a higher *D*_e_ and thus σ_e_ for the Little Broadhurst population. Removing this population did not change our results substantially. Using σ_e_, the median dispersal neighborhood was therefore a circle of radius 204 m (IQR: 170 to 280) (Fig. 3C). We also used σ_e_ to calibrate a Laplacian dispersal kernel (Fig. 3A) and obtained a median dispersal distance equal to 72 m (IQR: 60 to 101).

**Table 1.** Dispersal distance differs by orders of magnitude between the brooding coral *Stylophora pistillata* and the spawning coral *Pocillopora verrucosa*. For both species, we report the estimated neighborhood sizes (*NS*) in number of coral colonies, the estimated median axial dispersal distance ( σ ) in meters [measured using either an effective population density estimate ( σ_e_) or a census population density estimate ( σ_c_)], and the estimated median dispersal distance under a Laplace distributed kernel measured using σ_e_. Interquartile ranges are reported in brackets.

Species	*NS*	σ_e_	σ_c_	Laplace dispersal distance
*S. pistillata*	318 (286–356)	102 (85–140)	23 (22–25)	72 (60–101)
*P. verrucosa*	66E06 (54 × 10^6^ – 78 × 10^6^)	21,000 (18,000–24,000)	52,000 (47,000–57,000)	15,000 (13,000–17,000)

For *P. verrucosa,* we calculated genetic and geographic distances between colonies at the total range level with a 1D IbD model. We chose a 1D model because there were no signals of population structure range-wide and no signals of IbD at smaller spatial extents (Supplementary Materials). We obtained a median regression slope of 1.58 × 10^8^ (95% CI: 7.02 × 10E^−09^ to 2.46 × 10^−08^) (fig. S5), which was consistent across different levels of missing data (fig. S14) and estimated a median *NS* of 66 × 10^6^ colonies (IQR: 54 × 10^6^ to 78 × 10^6^). Using census counts for each site (table S6), we estimated a median census linear density *D*_c_ of 0.0060 colonies/m (IQR: 0.0059 to 0.0062). Using contemporary *N_e_* estimates for the southern and northern regions (table S5), we estimated a median linear effective density *D_e_* of 0.038 colonies/m (IQR: 0.030 to 0.048). Using these density estimates, we calculated a median axial dispersal distance σ equal to 52,000 m using *D*_c_ (IQR: 47,000 to 57,000) and 21,000 m using *D*_e_ (IQR:18,000 to 24,000) (Table 1). Using σ_e_, the median dispersal neighborhood was therefore a circle of radius 42,000 m (IQR: 36,000 to 48,000) (Fig. 3D). We also used σ_e_ to calibrate a Laplacian dispersal kernel (Fig. 3A) and obtained a median dispersal distance equal to 15,000 m (IQR: 13,000 to 17,000).

To corroborate dispersal distance estimates obtained using IbD analyses, we used COLONY (*49*) to identify probable pairs of kin colonies. For *S. pistillata*, we performed parentage analyses for each population separately and found a total of 28 pairs of colonies: 7 parent-offspring pairs, 1 full sibling pairs, and 20 half sibling pairs. For all pairs, the colonies were from the same reef, and for 20 of 28 pairs, the colonies were from the same site. Geographic distances between parent and offspring varied between 10 and 3600 m, the distance between the full siblings was 100 m, and the distance between half siblings varied between 10 and 2700 m (table S7). For *P. verrucosa*, COLONY did not identify any close kin.

### Levels of long-term gene flow among reefs vary between the brooder *S. pistillata* and the spawner *P. verrucosa*

**Fig. 4.**
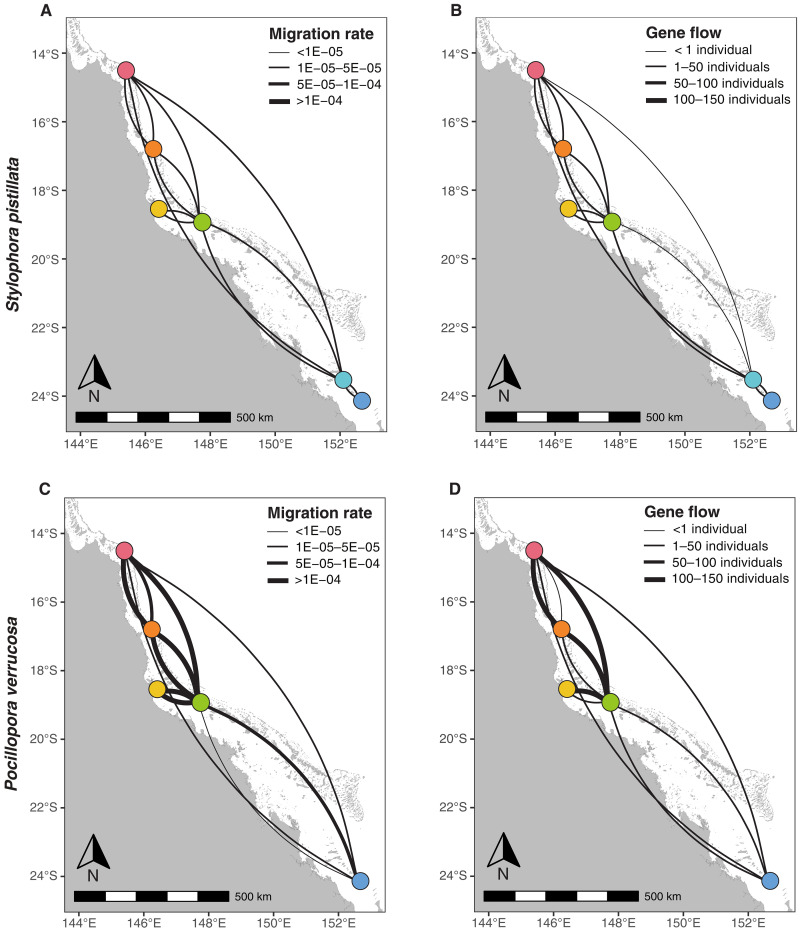
Migration rates and gene flow vary among *S. pistillata* and *P. verrucosa* populations across the GBR. Migration rates and gene flow inferred from models of population divergence with asymmetrical gene flow in δaδi, among six *S. pistillata* populations (**A** and **B**) and among five *P. verrucosa* populations (**C** and **D**). For all panels, arcs should be read clockwise to tell the directionality of migration rates or gene flow; the line thickness is proportional to the migration rates or gene flow. Reefs are color coded to match Fig. 1.

We optimized demographic models in the software tool δaδi (*20*) to jointly estimate long-term gene flow and population sizes between representative subsets of population pairs. For both species, we used a model of asymmetrical gene flow. In addition, for *P. verrucosa*, we tested the standard neutral model of panmixia, which had significantly lower support (based on Akaike information criterion and log likelihoods) than the asymmetrical gene flow model. We found convergence for all population pairs, with minimal residuals between the data site frequency spectra and the model site frequency spectra (figs. S6 and S7). After conversion of the δaδi parameters (Supplementary Materials; tables S3 and S4), we found that, on average, *S. pistillata* had lower migration rates (i.e., *m*) and smaller long-term *N_e_*, resulting in lower gene flow (i.e., *N_e_m*), compared to *P. verrucosa* (Fig. 4 and Table 2). For *S. pistillata*, migration rates varied from 1.09 × 10^−05^ (Lizard to Heron) to 5.45 × 10^−05^ (Moore to Lizard) and gene flow varied from 0.35 migrants per generation (Lizard to Heron) to 3.45 (Moore to Lizard). For *P. verrucosa*, migration rates varied from 4.28 × 10^−06^ (Davies to Lizard) to 1.11 × 10^−04^ (Davies to Pelorus) and gene flow varied from 0.77 migrants per generation (Moore to Lizard) to 136 (Lizard to Davies). Pearson’s correlation coefficient between gene flow and migration rates was 0.53 for *S. pistillata* and 0.59 for *P. verrucosa* (fig. S8). For both species, gene flow and migration rates to and from the southernmost reefs (Heron and Lady Musgrave) were the lowest. For *P. verrucosa*, migration rates were highly asymmetric and generally higher from north to south. Demographic modeling also revealed much larger long-term *N_e_* estimates for both species compared to contemporary *N_e_* estimates, according to expectations (*50*). Long-term *N_e_* ranged from 4950 (Pelorus population) to 7,787,000 colonies (Davies population) for *S. pistillata* and from 91,000 (Lady Musgrave population) to 16,500,000 colonies (Davies population).

**Table 2.** *S. pistillata* and *P. verrucosa* from the GBR have different population sizes, migration rates, and gene flow. We report geographic distances between populations (in meters) and parameter estimates from forward in time modeling of population divergence with asymmetrical gene flow using δaδi: population sizes *N*_e1_ and *N*_e2_ (in number of individuals), per-generation migration rate from population2 to population1 and from population1 to population2 (m_12_ and m_21_, respectively), and number of migrants per generation from population2 to population1 and from population1 to population2 (*N*_e_m_12_ and *N*_e_m_21_, respectively). Populations are ordered from the geographically closest together to the furthest away, with population1 north of population2. LM, Lady Musgrave.

Species	Populations	Distance	*N*_e1_	*N*_e2_	*M*_12_ S➔N	*M*_21_ N➔S	*N*_e_m_12_ S➔N	*N*_e_m_21_ N➔S
*S. pistillata*	Heron-LM	70,000	45,400	50,200	1.85 × 10^−05^	3.21 × 10^−05^	0.83	1.61
	Pelorus-Davies	120,000	4,950	120,000	4.13 × 10^−05^	2.52 × 10^−05^	2.05	3.02
	Moore-Davies	260,000	119,000	114,000	2.46 × 10^−05^	2.30× 10^−05^	2.93	2.63
	Lizard-Moore	260,000	63,200	147,000	5.45 × 10^−05^	1.39 × 10^−05^	3.45	2.05
	Lizard-Davies	520,000	97,400	115,000	2.46 × 10^−05^	1.28 × 10^−05^	2.39	1.47
	Davies-Heron	680,000	98,600	22,300	2.27 × 10^−05^	2.64 × 10^−05^	2.24	0.59
	Lizard-Heron	1,200,000	133,000	32,600	1.25 × 10^−05^	1.09 × 10^−05^	1.67	0.35
*P. verrucosa*	Pelorus-Davies	120,000	204,000	1,042,000	1.11 × 10^−04^	1.12 × 10^−04^	23	127
	Moore-Davies	260,000	203,000	764,000	1.18 × 10^−04^	1.27 × 10^−04^	23.9	96.9
	Lizard-Moore	260,000	1,650,000	731,000	8.74 × 10^−05^	1.06 × 10^−05^	144	0.77
	Lizard-Davies	520,000	353,000	352,000	4.28 × 10^−06^	1.11 × 10^−04^	15.0	136
	Davies-LM	750,000	742,000	144,000	9.48 × 10^−06^	7.05 × 10^−05^	7.0	10.2
	Lizard-LM	1,300,000	845,000	91,000	1.34 × 10^−05^	8.38 × 10^−05^	11.3	7.6

### Reduced and more variable within-population genetic diversity in the brooder *S. pistillata* compared to the spawner *P. verrucosa*

To investigate the extent of genetic diversity within populations (defined as all colonies sampled at one reef), we used six genetic diversity statistics (Table 3). We only included populations with more than eight colonies, thus excluding *P. verrucosa* populations from Heron, Lady Musgrave, Flinders, Dungeness, and Masig (tables S1 and S2). Autosomal nucleotide diversity (π) estimates, calculated using both variant and invariant sites using PIXY (*51*), were similar in both species, around 1% nucleotide differences, with little variation among populations. Autosomal expected heterozygosity (*H*_e_) estimates, calculated using both variant and invariant sites in Stacks (*52*), were substantially higher in *P. verrucosa* (between 0.082 and 0.084) compared to *S. pistillata* (between 0.037 and 0.059). *H_e_* estimates were similar among all *P. verrucosa* populations but varied among *S. pistillata* populations, with Heron, Lady Musgrave, and Pelorus populations having substantially lower values (Table 3). Rarefied allelic richness estimates were higher in *P. verrucosa* (between 1.44 and 1.48) compared to *S. pistillata* (between 1.18 and 1.28) and more variable among *S.* pistillata populations, with the lowest values for Heron, Lady Musgrave, and Pelorus populations. Using the ADZE program (*53*), we found no private alleles in *P. verrucosa* compared to small numbers in all *S. pistillata* populations. Contemporary effective population sizes (*N*_e_) estimated using the LD method in NeEstimator (*54*) were variable among *S. pistillata* populations (ranging from 51 to 964), with the smallest populations at Lady Musgrave, Pelorus, and Chicken reefs. Contemporary *N*_e_ estimates did not correlate with reef area. For *P. verrucosa*, we estimated contemporary *N*_e_ at various spatial extents and the smallest extent at which we could obtain non-infinite estimates was at the regional level. *N*_e_ was estimated at 31,000 colonies for the southern populations and at 43,000 for the northern populations. With lower levels of missing data, *N*_e_ estimates decreased (table S8), consistent with previous empirical studies investigating the effect of missing data on *N*_e_ estimation (*55*, *56*). Last, inbreeding coefficients (*F*_IS_) were marginally and nonsignifically (*t* test *P =* 0.0002) negative for all populations of both species, suggesting slight excesses of heterozygotes.

**Table 3.** Genetic diversity within *P. verrucosa* and within *S. pistillata* populations. Metrics include mean autosomal nucleotide diversity (π), autosomal expected heterozygosity (*H*_e_), mean allelic richness (AR), number of private alleles (PA), effective population size (*N*_e_), and inbreeding coefficient (*F*_IS_). *N* is the number of individuals per population. Only populations with *N* ≥ 8 were included. *N*_e_ for *P. verrucosa* was calculated at the regional level instead of reef level and was estimated at 31,000 individuals for the southern region and 43,000 individuals for the northern region.

Species	Population	N	π	*H_e_*	AR	PA	*N_e_*	F_IS_
*S. pistillata*	Heron	22	0.0112	0.0399	1.18	0.006	423	−0.011
	Lady Musgrave	8	0.0106	0.0374	1.18	0.02	51	−0.0078
	Davies	81	0.0104	0.0504	1.26	0.03	964	−0.019
	Chicken	13	0.0104	0.0516	1.25	0.04	131	−0.012
	Little Broadhurst	21	0.0104	0.0475	1.26	0.04	854	−0.012
	Pelorus	21	0.0106	0.0386	1.22	0.03	114	−0.010
	Moore	34	0.0104	0.0593	1.25	0.04	594	−0.020
	Lizard	32	0.0105	0.0516	1.26	0.05	251	−0.015
*P. verrucosa*	Davies	75	0.0104	0.0839	1.48	0	-	−0.012
	Chicken	26	0.0104	0.0838	1.44	0	-	−0.012
	Little Broadhurst	22	0.0104	0.0824	1.44	0	-	−0.017
	Pelorus	30	0.0106	0.0848	1.46	0	-	−0.014
	Moore	34	0.0104	0.0818	1.46	0	-	−0.011
	Lizard	23	0.0105	0.0828	1.45	0	-	−0.012

## DISCUSSION

Despite the rapid decline of reef-building corals globally, the ecological and evolutionary processes that are critical to their persistence are still not well understood. Our study demonstrates that realized dispersal distances and gene flow rates can vary greatly between co-distributed coral species. These findings reveal extreme variation in connectivity among corals and in turn imply different demographic dynamics, adaptive capacities, and spatial distributions of genetic diversity. With substantial sampling across the GBR and genome-wide sequencing data, we show that the brooding species *S. pistillata* disperses across meters each generation, with little gene flow among reefs. In contrast, the spawning species *P. verrucosa* disperses across tens of kilometers, with sufficient gene flow among reefs to prevent the accumulation of large allele frequency differences (*F*_ST_ < 0.048). Our results highlight that the spatial extents of dispersal and gene flow can vary greatly among coral species with different reproductive modes and yield quantitative estimates that can guide monitoring, conservation, and restoration programs.

### Dispersal distances differ greatly between brooding and broadcast spawning coral species

Coral larval dispersal is a critical process for coral reef ecosystem trajectories. Here, we estimated axial dispersal distances (σ, hereafter referred to as dispersal distances) using isolation by distance (IbD) models, calibrated independently with census (*D*_c_) and effective (*D*_e_) population densities. IbD analyses revealed localized generational dispersal in *S. pistillata*, with estimated median dispersal distances of 23 (σ_c_) and 102 (σ_e_) m (Table 1 and Fig. 3). Parentage analyses identified 28 kin pairs with all close relatives inhabiting the same reef, in agreement with the IbD results. Meter-scale dispersal distances, albeit smaller, have previously been estimated in other brooding coral species: between 2 and 11 m in *Agaricia* corals (*28*) and between 3 and 4 m in *Pocillopora damicornis* (*26*), aligned with previous reports of short larval duration in most brooding coral species. These short dispersal distances estimated across studies imply that larval retention and local recruitment within reefs are common in brooding corals.

Theory predicts a population to be isolated when separated by more than 2σ from any other population due to too few individuals immigrating at this distance (*57*). Following this prediction, we expect high population connectivity of brooding coral populations within reefs. In addition, in the absence of demographically important external larval supply from other reefs (that are often kilometers apart), populations of brooding corals are likely ecologically isolated and primarily self-reliant. Thus, recovery of brooding corals after a local disturbance event will greatly depend on the number (and density) of survivor colonies at the local scale. As a result, we might expect more rapid local repopulation of brooding species after low-mortality events, while high-mortality events such as catastrophic storms or mass bleaching events might put brooding populations at higher risk of local extinction. *S. pistillata* was found to have higher mortality rates compared to *P. verrucosa* during the 2016 mass bleaching event on the GBR (*58*), and data from the AIMS Long Term Monitoring Program (*59*) show a general decline of *S. pistillata* across the GBR over the past 20 years, including at our sampling sites (fig. S11).

Contrasting with findings of meter-scale dispersal in *S. pistillata*, we estimated median dispersal distances of 21 (σ_e_) and 52 (σ_c_) km in *P. verrucosa* (Table 1 and Fig. 3). These large distances are consistent with experimental evidence for longer pelagic larval duration in spawning coral species [reviewed in (*60*)]. However, our results contrast with biophysical models that often estimate dispersal distances of approximately 100 km for spawning corals [e.g., (*61*, *62*)] more than five times our effective median estimate for *P. verrucosa*, supporting a recent call for the use of higher resolution biophysical models to more accurately capture dispersal patterns (*63*). In addition, biophysical models represent potential dispersal, whereas IbD models capture realized dispersal (movement, settlement, and reproductive success). On the GBR, distances between reef systems vary, which will dictate the level of population isolation in *P. verrucosa* (*57*). If two reefs are less than 2σ (i.e., 40 to 100 km) apart, then a substantial number of larvae will frequently disperse between reefs, and populations would therefore be less reliant on local retention dynamics. If reefs are more than 2σ apart, then populations will be isolated on ecological timescales. Where larval exchange naturally occurs between reefs, this high connectivity will likely aid replenishment following a disturbance event (*64*, *65*). This potential for longer dispersal in *P. verrucosa* also indicates a higher potential for tracking optimal climatic niches via range shifts (*66*).

Our study adds to the growing body of literature showing that IbD theory can successfully be used to evaluate contemporary dispersal distances in marine species (*28*, *67*, *68*). This method is limited, however, by its assumption of constant population density across space. Specifically, there is no comprehensive theory for how realistic landscapes affect IbD-derived estimates, such as landscapes with areas of both continuous and patchy habitats (*57*). In addition, although we can estimate a mean dispersal distance, whether there is suitable habitat at this distance will dictate the success of any dispersal event. Thus, IbD-derived estimates are averaged across the sampling range but will in reality vary depending on population density and habitat availability. Rather than focus on exact quantitative estimates, we stress that the relative magnitudes of contemporary dispersal distances among the two species (i.e., tens of meters versus tens of kilometers) are likely to be real in the context of the GBR seascape and that similar contrasts may be observed between other brooding and broadcast spawning species for the GBR and other seascapes.

### Asymmetrical gene flow between coral populations vary substantially among species

Long-term gene flow is the key parameter shaping spatial patterns of genetic variation across a species’ range and over many generations (*9*). Using demographic modeling, we found limited gene flow between distant *S. pistillata* populations, with *N_e_m* < 5, consistent the maintenance of reef-scale population structure (Figs. 2 and 4). As a result, long-distance dispersal events are likely too rare to be ecologically relevant. In comparison, *P. verrucosa* populations are historically well connected, with higher gene flow, explaining limited allele frequency differences between populations occupying reefs separated by up to hundreds of kilometers (e.g., *F*_ST_ = 0.018, between Lizard and Heron) (Figs. 2 and 4). Migration rates between *P. verrucosa* populations were strongly asymmetrical, with generally more extensive migration from northern populations to southern populations (Fig. 4). This is consistent with the directionality of oceanic currents, flowing from north to south along the eastern coast of Australia and with previously inferred migration patterns among populations of the spawning corals *Acropora millepora* (*23*) and *Acropora tenuis* (*21*) across the GBR.

In addition, we note that for both species, migration rates (the proportion of migrants, *m*) were more stable than gene flow (the number of migrants, *N_e_m*) and of similar magnitude between species. This highlights the important role of population sizes in modulating gene flow: Larger populations will produce more potential propagules and therefore export a greater absolute number of migrants than a smaller population with equal migration rates (*69*). Differences in fecundity or reproductive investment between species might also play a role in the gene flow rate differences inferred. While broadcast spawners make more larvae than brooders and therefore more propagules, brooded larvae might have better survival chances (*70*, *71*). However, effective migration and gene flow rates are a summation of reproductive traits, dispersal, and survival, and these cannot be teased apart with only two species under examination. Another important source of uncertainty affecting migration rate estimates is the mutation rate (but generation time is not). Specifically, a higher mutation rate would result in higher migration rate estimates (Supplementary Materials). While obtaining direct estimates of mutation rates in coral species would greatly improve such analyses, our between-species comparative conclusions remain sound.

### Gene flow among coral populations shape the spatial distribution of genetic diversity

Gene flow across a species’ range influences the distribution of standing genetic diversity within and between populations, which represents the raw material for natural selection. Measuring the extent and spatial distribution of genetic diversity is therefore important for predicting a species’ evolutionary potential. Although we might expect substantial differences in measures of total genetic diversity among reefs, values were fairly consistent for both species (Table 3). However, the three marginal populations for *S. pistillata* consistently showed lower genetic diversity: Heron and Lady Musgrave, two reefs on the more isolated southern edge of the GBR, and Pelorus, a turbid-water reef in the inshore edge of the central GBR. This follows theoretical predictions of lower neutral genetic diversity in range edge populations due to increased isolation, founder effects, or increased environmental selection. These results also match field observations reporting that *S. pistillata* is more common at northern and offshore reefs of the GBR (*72*). Genetic diversity was higher and more evenly distributed in *P. verrucosa* populations (Table 3) likely due to extensive gene flow homogenizing allele frequencies among reefs. This suggests that adaptation from standing genetic variation might be easier in this species. For *S. pistillata,* within-population diversity was lower, consistent with strong local genetic drift. However, range-wide genetic diversity was higher, reflected by a higher number of private alleles, as expected for species with high population structure. Thus, higher rates of local adaptation are anticipated for this species.

Effective population size (*N_e_*) is another measure commonly used as a proxy for population adaptive potential, but different approaches available to estimate this parameter either represent different time periods or concepts (*50*). In this study, we inferred the effective number of breeders (*NS*) within the dispersal neighborhood (using IbD models), contemporary *N_e_* based on linkage disequilibria (using NeEstimator), and long-term *N_e_* (using demographic modeling in δaδi). Contemporary *N*_e_ is relevant to the last few generations and depend on the geographic locations of the sampled individuals. It is conceptually the same as *NS* (*73*). Long-term *N_e_* estimates are relevant for longer time periods and represent coalescent events across a larger spatial expanse. Thus, it is expected to find greater long-term *N_e_* values in both species (Tables 1 and 2). For conservation purposes, estimates of contemporary *N*_e_ or *NS* are more useful as they are representative of the last few generations (*74*). For *S. pistillata*, similar contemporary *N*_e_ and *NS* estimates suggest that contemporary *N*_e_ was estimated at the spatial extent of the breeding window (*73*). For *P. verrucosa*, however, we found *NS* to be much larger than contemporary *N*_e_. This is likely due to pooling overly genetically divergent colonies for the contemporary *N*_e_ calculations (Table 1), which can create mixture LD (*75*, *76*) and downwardly bias *N*_e_ (*73*). This could unfortunately not be avoided, given the inability to measure contemporary *N*_e_ at smaller spatial extents. Robust estimation of contemporary *N*_e_ for large-population marine species is a known challenge (*77*), and these estimates suffer from greater uncertainty. With this bias in *P. verrucosa N_e_* in mind, it is more useful to compare species based on *NS* values. With a much greater number of breeding colonies in *P. verrucosa* (*NS* = 66 × 10^6^ colonies) compared to *S. pistillata* (*NS* = 318 colonies), genetic drift is undoubtedly weaker in *P. verrucosa*, leading to a slower loss of genetic variability each generation at the scale of the genetic neighborhood.

In addition, we also estimated census population sizes (*N*_c_) and can therefore compare these independently derived values to contemporary *N*_e_ estimates. In general, *N*_e_ is expected to be considerably less than *N*_c_ (and correspondingly, *D*_e_ < *D*_c_), because not all individuals will contribute to the next generation (*78*). The ratio *N*_e_/*N*_c_ is of particular interest for management and conservation because if this ratio is stable across taxa, it can be used to infer *N*_c_ from *N*_e_ or vice versa (*79*). For *S. pistillata,* the *D*_e_/*D*_c_ ratio was 0.05, which is comparable to *N*_e_/*N*_c_ ratios for other marine species (*77*). For *P. verrucosa*, the *D*_e_/*D*_c_ ratio was 6.3, much greater than expected. This high value could be the result of a biased *D*_c_ estimate, measured as an approximate linear density (which is more of an abstract mathematical concept; see Materials and Methods) and extrapolated from census counts at a limited number of sites to a larger area. Verification with more abundance data across the GBR is therefore required to get a more accurate estimate of *D*_c_. In addition, *D*_c_ and *D*_e_ were calculated at different scales for *P. verrucosa*: *D*_e_ was estimated from *N*_e_ calculated in NeEstimator at the regional scale, whereas *D*_c_ was estimated from *N*_c_ extrapolated from count data at the plot level. An accurate comparison would be to measure both *N*_c_ and *N*_e_ at the scale of the neighborhood size, but this was not possible here because of sample sizes (see Materials and Methods). Alternatively, we can also speculate that *N*_c_ < *N*_e_ is a true observation for *P. verrucosa*, which could be explained by a recent decrease in census numbers and a lag in *N*_e_ reduction due to large population sizes. These results emphasize the difficulties in generalizing *N*_e_/*N*_c_ ratios across taxa with different life histories (*80*). Nevertheless, despite the uncertainty around *N*_e_ and *N*_c_ estimates, the results contrasting genetic diversity and connectivity between *S. pistillata* and *P. verrucosa* are very consistent.

### Connectivity dynamics affect coral species’ adaptative potential

Differences in dispersal capacities, gene, flow and genetic diversity between co-distributed coral species, such as we observe in this study, set the stage for different adaptive dynamics. Although gene flow differed significantly between *P. verrucosa* and *S. pistillata*, migration rates did not. While gene flow determines the extent of population differentiation, the evolution of local adaptation depends on the migration rates (*11*, *12*, *81*). Here, somewhat counterintuitively, similar migration rates between species suggest that they have similar selection coefficient thresholds and that local adaptation can develop over similar spatial extents. Coefficients of selection pressures relevant to corals in nature are currently not quantified. Further research is therefore needed to know whether the migration rates estimated here would prevent or facilitate local adaptation. In addition, greater dispersal capacities are expected to favor phenotypic plasticity (*82*), which could explain the broad range of morphologies reported for *P. verrucosa* (*83*).

For *S. pistillata*, while differentiated populations may be more locally adapted to their reef environment, the likelihood of “preadapted” heat tolerance alleles naturally being transported from one reef to another via migration, at a pace matching current ocean warming, is very small. Combined with lower levels of within-reef standing genetic variation, the low likelihood of naturally occurring evolutionary rescue makes *S. pistillata* populations more at risk for becoming maladapted as environmental conditions change (*84*, *85*). Consequently, *S. pistillata* and other brooders might be good candidates for active restoration actions such as assisted gene flow and assisted migration (to extend the species range) (*86*). Moving coral colonies or gametes from Northern GBR to Southern GBR populations has been proposed to increase the frequency of warm-adapted alleles in the target populations (*43*). This could potentially facilitate adaptive gene flow for brooding corals and provide demographic support if the target populations have small effective sizes (*86*). However, the introduction of new alleles into small and strongly locally adapted recipient populations could increase genetic load (*87*), so considering both the benefits and risks associated with assisted gene flow is critical.

In contrast, high levels of gene flow among *P. verrucosa* populations increase global adaptation potential at the scale of the species range. With climates shifting to warmer states, the extent and directionality of gene flow are important for the spread of warm-adaptive alleles and to prevent coral population maladaptation, especially for range edge populations (*88*, *89*). With increased natural migration rates from Northern to Southern GBRs, the risk of population maladaptation, including in the Southern GBR, might be reduced for *P. verrucosa*. As a result, this species is unlikely to benefit greatly from assisted migration or assisted gene flow (*86*).

In conclusion, the spatial extent of connectivity via dispersal and gene flow can differ by orders of magnitude among co-distributed coral taxa, with consequences for the extent and geographic partitioning of genetic diversity. Our empirical estimates offer insights to better predict the recovery of coral populations following local disturbances and their adaptive potential facing climate change. Future studies are needed to verify the consistency of these connectivity patterns across different seascapes and to test whether reproductive modes can be used to make general inferences about coral species dispersal.

## MATERIALS AND METHODS

### Study species

*S. pistillata* (Esper, 1792) and *P. verrucosa* (Ellis and Solander, 1786) were chosen as the two target species for this study. They are both abundant, sympatric on the GBR, and closely related (family Pocilloporidae) but differ in their reproductive mode. These attributes make them an ideal pair to investigate coral connectivity patterns and processes, as environmental and phylogenetic biases will be minimized (*90*). *S. pistillata* produces large, brooded larvae that settle within a few hours to a few days after being released in experimental conditions (*91*–*93*). On the GBR, previous population genetics studies on *S. pistillata* have reported contrasting results: lack of population structure using nuclear and mitochondrial markers (*94*) and high population differentiation using allozymes (*30*). On the GBR, *P. verrucosa* is described as a broadcast spawner (*95*), but its reproductive traits and population genetic attributes have not yet been investigated.

### Field work and sampling design

Adult colonies of *S. pistillata* and *P. verrucosa* were sampled on SCUBA (permit number G21/44774.1) from 8 and 12 reefs, respectively, of the GBR. At each reef, we sampled at multiple sites and depths (tables S1 and S2 and fig. S10). Study sites spanned over 1000 km in latitude and inshore-offshore gradients covering four ecologically meaningful scales: (i) reef clusters (100s km), (ii) reefs within clusters (10 to 100s km), (iii) habitats within reefs (1 to 10s km), and (iv) depths within habitats: shallow (3 to 8 m) and deep (9 to 15 m). Each site consisted of four 72 m^2^ semipermanent “plots” laid parallel to the depth gradient and within an overall surveyed area of ~1500 m^2^ (*96*). This nested sampling design aimed to capture representative habitats across geographic locations. At each study site, we haphazardly sampled coral colonies that were at least 3 m apart. Small fragments, between 1 and 3 cm^2^, were preserved in 100% ethanol.

For each plot, photogrammetry (i.e., high-quality imaging of the reef) was performed to create high-resolution orthomosaics (i.e., 2D models). Detailed methods describing photogrammetry image capture and model processing are included in the Supplementary Materials. Digitization of all *S. pistillata* and *P. verrucosa* colonies identified on the orthomosaics was conducted in the software TagLab (*97*) by three experienced benthic ecologists. Species identification accuracy was 98% for *S. pistillata* and 90% for *P. verrucosa* from in situ validation from an experienced taxonomist.

### Cryptic species delineation

Cryptic taxa are a recurrent finding in coral population genetics studies (*37*, *98*). Thus, we screened and filtered our datasets to ensure that we only included colonies forming cohesive genetic groups. For *S. pistillata*, we used previously published data from (*46*), which found five genetically distinct taxa within the nominal species across the GBR in a dataset of 379 samples collected from 70 sites across 11 reefs. In the present study, we only included the 232 samples identified as *S. pistillata* taxon1 from (*46*), collected from 46 sites across eight reefs. For newly sampled *P. verrucosa* colonies, we performed preliminary clustering analyses on a larger dataset consisting of 423 samples, collected from 80 sites across 17 reefs, that were identified as either *Pocillopora damicornis*, *Pocillopora acuta*, *Pocillopora meandrina*, *Pocillopora* eydouxi, or *P. verrucosa* in the field. PCA revealed several genetic clusters, not always matching the morphospecies assignment (fig. S9). In this present study, we focused on one cohesive genetic group of 224 samples, collected from 49 sites across 12 reefs, that were mostly identified as *P. verrucosa* in the field, including colonies verified as *P. verrucosa* by a taxonomist using skeletal fragments and field photographs.

### DNA extraction, library preparation, and sequencing

We extracted genomic DNA using the DNeasy QIAGEN kit according to the manufacturer’s instructions, and we ran agarose electrophoresis gels to visually assess the quality of the DNA extractions. For both species, we prepared ddRAD libraries. For *S. pistillata*, libraries were prepared using the *MspI* and *PstI* enzymes and the protocol outlined in (*99*) [refer to (*46*)]. For *P. verrucosa*, ddRAD libraries were prepared at the Australian Genome Research Facility (AGRF) using the *EcoRI* and *HpyCH4IV* enzymes and selection of fragments between 280 and 375 base pairs. For both library sets, sequencing was performed on NovaSeq SP 300 lanes at AGRF.

### Genomic data filtering

We used IPYRAD (*100*) to demultiplex, cluster, and align reads using standard parameters, for both datasets independently. We used contig-level reference genomes to map reads for both the *S. pistillata* (GenBank genome assembly APGP_CSIRO_Spis_v1, accession number PRJNA1000647) and *P. verrucosa* (GenBank genome assembly CSIRO_AGI_Pver_v1, accession number PRJNA1000218) datasets. After screening for cryptic species, we selected individuals belonging to the two species of interest and recalled variants in IPYRAD. The datasets were further filtered using VCFTools (*101*). First, we filtered for biallelic sites, a minimum allele count (MAC) of 3, a minimum read depth of 5, a maximum read depth of 100, and a maximum of 50% missing data per site. Then, we filtered out samples with large amounts of missing data using a 30% threshold for the *S. pistillata* dataset and a 50% threshold for the *P. verrucosa* dataset. In addition, we identified clonal pairs using the vcf_clone_detect.py script from P. Bongaerts (https://github.com/pimbongaerts/radseq), which identifies clonal pairs based on a break in the distribution of pairwise allelic similarity among all samples. We kept one sample per clonal pair, choosing the colony with the least amount of missing data. Each species dataset was then filtered to allow a minimum allele frequency (MAF) of 0.01 and a maximum threshold for missing data per site, using a 5% threshold for the *S. pistillata* dataset and a 20% threshold for the *P. verrucosa* dataset. This more lenient missingness threshold was used for the *P. verrucosa* dataset to retain a sufficient number of SNPs to obtain reliable *N_e_*, IbD slope, demographic, and diversity estimates. We have also applied 15 and 5% missingness thresholds to the *P. verrucosa* dataset to explore how downstream analyses would be affected and report results in the Supplementary Materials. Last, SNPs in physical linkage were removed using PLINK (*102*) with a sliding window of 50 SNPs, 5 SNPs to shift window and a variance inflation factor threshold of 2.

### Population structure analyses

To infer range-wide population structure, we used two different approaches. First, to visualize the partitioning of genetic variation among samples, we performed a PCA using the “adegenet” R package (*103*). For the *S. pistillata* dataset, we used the “MCMCpack” R package (*104*) to apply a Procrustes transformation to the first PC axes and rotated them to match spatial coordinates for graphical purposes. Second, we performed a clustering analysis in ADMIXTURE (*47*) to assign samples to genetic groups using a maximum-likelihood approach. We explored *K* = 2 to *K* = 15 and chose the optimal *K* having the lowest cross-validation error and highest log likelihood (*105*). To estimate population differentiation, we calculated pairwise *F*_ST_ between populations using the “dartR: R package (*106*).

### Genetic diversity analyses

To investigate genetic diversity within populations (i.e., alpha diversity), we used six metrics calculated at the reef level for both species: mean nucleotide diversity, expected heterozygosity, inbreeding coefficient, mean allelic richness, number of private alleles, and contemporary effective population size. Omission of invariant sites can bias measures of nucleotide diversity (*51*) and heterozygosity (*107*). To avoid this, we created VCF files containing both variant and invariant sites for each dataset. First, we used BCFtools mpileup (*108*) to generate an all-site VCF file, which we filtered to remove indels, and apply minimum depth, maximum depth, and missing data thresholds (same thresholds described above). Then, we separated variant and invariant sites and filtered the variant sites for MAC and MAF (the same as above). To calculate mean nucleotide diversity, we used PIXY with a window size of 1000 (*51*). We combined information across windows following the authors’ recommendations. To calculate expected heterozygosity, we used the Stacks program “Population” (*52*) and ran each population separately with no missing data, following the recommendations in (*107*). We obtained inbreeding coefficient estimates from the Stacks output file. Using only populations with more than eight samples, we used the “PopGenReport” R package (*109*) to calculate allelic richness, which uses rarefaction to account for sample size differences. To calculate the number of private alleles, we used the ADZE program, which also uses rarefaction to account for sample size differences (*53*). Last, contemporary effective population sizes (*N_e_*) were obtained using the LD method in NeEstimator (*54*). We measured reef-level *N_e_* estimates for *S. pistillata* and regional-level *N_e_* estimates for *P. verrucosa* (see Supplementary Materials for details).

### Isolation by distance and estimation of dispersal distances

To evaluate the decline in dispersal probability at increasing geographic distance, we used isolation by distance (IbD) theory, which predicts a positive relationship between genetic and geographic distance among individuals (*15*). IbD models assume isotropic dispersal, which is probably a reasonable assumption for short-distance dispersal events of marine species. IbD regressions were performed in the genepop R package (*48*), using Rousset’s â as a metric of genetic distance between samples. Considering the spatial dependence of the data is important for IbD analyses (*110*). For *S. pistillata*, given the notable population structure among reefs, we performed the IbD regression at the reef scale (colonies separated by 0 to 10,000 m). At this spatial extent, the length of the habitat is not much greater (>99%) than the width, so we used a 2D IbD model (i.e., using log-transformed geographic distances), following recommendations in (*15*). For *P. verrucosa*, given the absence of population structure, we performed IbD regressions at different spatial extents. Only the regression including all pairs of samples was significant. At this spatial extent, the length of the sampling area (2000 km) is greater (>90%) than its width (120 km), so we used a 1D IbD model, following recommendations in (*15*). For both datasets, we used approximate Bayesian computation bootstrapping to obtain a 95% CI around the regression slope estimate.

For each dataset, we used the slope of the IbD regression (*b*) and estimates of population density (*D*, in number of individuals/m^2^ for a 2D model and in number of individuals/m for a 1D model) to estimate the mean parent-offspring axial dispersal distance (σ, in meters). We used Eq. 1 for *S. pistillata* (2D model) and Eq. 2 for *P. verrucosa* (1D model) (*14*, *15*)

$$\sigma =\sqrt{\frac{1}{4D\mathrm{b\pi}}}\;$$ (1)

$$\sigma =\sqrt{\frac{1}{4Db}}$$ (2)

Then, we estimated the neighborhood sizes (*NS*, in number of individuals), which corresponds to the effective number of adults within twice the mean axial dispersal distance. We used Eq. 3 for *S. pistillata* (2D model) and Eq. 4 for *P. verrucosa* (1D model) (*14*, *15*)

$$\mathrm{NS}=4D\pi \;\sigma^{2}$$ (3)

$$\mathrm{NS}=4D\;\sigma^{2}$$ (4)

To obtain density estimates, we used both census population sizes (*N_c_*) and effective population sizes (*N_e_*) to yield census population densities (*D_c_*) and effective population densities (*D_e_*), respectively. Details of population size and density calculations can be found in the Supplementary Materials.

We propagated uncertainties in each parameter estimate by resampling the parameters from appropriate probability distributions and creating a joint probability distribution of σ. Details can be found in the Supplementary Materials.

We used effective σ estimates to parameterize indicative dispersal kernels using a Laplacian distribution, which is described by p(*x*) = $\frac{1}{\sigma \sqrt{2}}e^{\frac{-\sqrt{2}x}{\sigma }},$ the probability that an individual disperses at a distance *x* away from its parent. Several other marine species have dispersal kernels that conform to the Laplacian distribution (*68*, *111*, *112*). The dispersal distance with a Laplacian kernel is $\frac{\sigma }{\sqrt{2}}$.

### Kinship analyses

In complement to the IbD analyses, we used COLONY v.2.7.0.1 (*49*), to identify probable pairs of parents-offspring, full siblings, and half siblings. We performed these analyses for each population separately for *S. pistillata* as COLONY assumes a sample of individuals taken from a randomly mating population (*49*). We created input files using the “radiator” R package (*113*), specifying a polygamous and monoecious mating system, as *S. pistillata* and *P. verrucosa* are described as hermaphroditic (*91*, *114*), and considered all samples as potential mothers, fathers, and offspring. We chose a false allele rate of 0.0045 after calculating a maximum genetic dissimilarity of 0.43% between the nine pairs of clonal samples (no sequencing of technical replicates). We arbitrarily chose a value of 0.01 for the probability of having sampled an offspring (but changing this value did not affect the results). For each dataset, we performed 10 runs of medium length with the full-likelihood method and high-likelihood precision. For each parent-offspring or sibling pair identified, we calculated the spatial distance between them using the “sf” R package (*115*).

### Estimation of long-term gene flow

To investigate the extent of evolutionary connectivity, we estimated long-term gene flow and migration rates as well as long-term effective population sizes between several population pairs using diffusion approximation of demographic inference (δaδi) (*20*). For both datasets, we used models of divergence with asymmetric gene flow, since asymmetric passive dispersal with oceanic current is often reported for marine larvae, including corals (*21*, *23*). For *P. verrucosa*, we additionally tested a neutral model of no population divergence for all population pairs, given weak population structure. For *S. pistillata*, we considered seven population pairs, separated by geographic distances ranging from 70 to 1200 km: Heron-Lady Musgrave, Pelorus-Davies, Moore-Davies, Lizard-Moore, Lizard-Davies, Davies-Heron, and Lizard-Heron. For *P. verrucosa*, we considered six population pairs, separated by geographic distances ranging from 130 to 1,300 km: Pelorus-Davies, Moore-Davies, Lizard-Moore, Lizard-Davies, Davies-Lady Musgrave, and Lizard-Lady Musgrave.

We generated folded joint allele frequency spectra (JAFS) for each population pair, masking singletons and doubletons due to their high sequencing error rates and filtering in previous steps. We followed the framework developed by K.P. (https://github.com/kepra3/kp_dadi) and searched the parameter space by performing three rounds of optimization with three, two and onefold permutations with at least 30 runs per round. We visually inspected the residuals between the model-fitted JAFS and the original data JAFS and used the Fisher information matrix, which is appropriate for unlinked data, to estimate parameter SDs.

To obtain estimates from δaδi outputs, a mutation rate and a generation time are needed. For the mutation rate, we used μ = 1.2 × 10^−8^ mutations per base per generation for both species, which has been estimated for Acroporidae corals based on sequence divergence estimates (*24*). No direct measures of generation time exist for corals, but time to first reproduction based on laboratory observations from field-collected fragments has been estimated to 2 years in both *S. pistillata* (*89*) and *P. verrucosa* (*116*). To account for uncertainty in these estimates and the fact that generation time is typically higher than time to first reproduction, we used a generation time of 3 years for both species. We acknowledge that these are approximate estimates, and our analyses could greatly benefit from more accurate, direct, measures. However, the δaδi relative estimates are reliable among populations and are also likely reliable among species if their mutation rates do not differ substantially. We then converted δaδi parameters into effective population sizes (ancestral population, *N*_ref_, and both populations, ν_1_ and ν_2_) as number of individuals, migration rates as fractions of new migrants per generation (m_12_ and m_21_), and gene flow rates as the number of migrant individuals per generation (N_e_m_12_ and N_e_m_21_) (Supplementary Materials).

## Supplementary Materials

### This PDF file includes:

Supplementary text Figs. S1 to S14 Tables S1 to S8 References
