# Supplementary material for: Connectivity differs by orders of magnitude among co-distributed corals, affecting spatial scales of eco-evolutionary processes
Source: Sci Adv. 2025 Jul 2;11(27):eadt2066. doi: 10.1126/sciadv.adt2066 (PMC12219506; doi:10.1126/sciadv.adt2066)
Supplement: Supplementary file 1 — Supplementary text Figs. S1 to S14 Tables S1 to S8 References [file sciadv.adt2066_sm.pdf]

Science Advances
AAAS

Supplementary Materials for

Connectivity differs by orders of magnitude among co-distributed corals, affecting spatial scales of eco-evolutionary processes

Zoe Meziere *et al.*

Corresponding author: Zoe Meziere, z.meziere@uq.edu.au

Sci. Adv. **11**, eadt2066 (2025)
DOI: 10.1126/sciadv.adt2066

This PDF file includes:

Supplementary text
Figs. S1 to S14
Tables S1 to S8
References

Supplementary text

Photogrammetry

Photogrammetry image capture and model processing techniques followed standardized workflows described in (*96*). In short, each plot was imaged using two Nikon D850 DSLRs with 20mm Nikkor lenses in Nauticam housings with 8" dome ports. All cameras were mounted on an aluminum frame spaced 57 cm apart. Prior to imaging, six ground control points were distributed across the depth gradient of each plot and their depth recorded to aide with scaling, orientation and incorporate bathymetry into 3D and 2D model-building.

SCUBA divers captured imagery using a 'lawn mower' swim pattern and shooting continuously at 0.5 second intervals at an altitude of ~1.0 m, achieving approx. 80 % overlap between images. Images were captured in between January and May 2021, for the first survey and recaptured on an annual basis.

Photogrammetry imagery was processed in Agisoft Metashape Professional v.1.7.6 (Agisoft LLC, St. Petersberg, Russia) using steps outlined detailed in (*96*). Three-dimensional (3D) models were scaled and oriented using GCPs. Resultant orthomosaics were oriented real world up, had an approximate resolution of 0.3 mm pixel and were exported at a maximum dimension of 3200 pixels.

Effect of missing data filtering in *Pocillopora verrucosa*

Due to poorer genotyping quality in the *P. verrucosa* dataset, we have decided to allow for higher missingness (< 20%) compared to the *S. pistillata* dataset (< 5%), to retain a sufficient number of SNPs for the analyses. To make sure that results hold true across different thresholds, we have repeated the PCA, pairwise-F_{ST} calculations, IbD regressions, and N_e estimations on two additional datasets, filtered to allow a maximum of 15% and 5% missing data, respectively. With < 20% missing data, we retained 8,597 SNPs, with < 15% missing data, we retained 3,808 SNPs we and with < 5% missing data, we retained 122 SNPs.

PCA were not affected by levels of missing data, with no discernible patterns of population structure at any level of missing data (fig. S12). Similarly, population pairwise F_{ST} values, which are good summary statistics to compare different Site Frequency Spectra, were not greatly affected by levels of missing data (fig. S13). We found that the maximum absolute population

pairwise F_{ST} value differences were 0.03 and 0.05, between the dataset with < 20% missing data and the datasets with < 15% and < 5% missing data, respectively.

IbD regressions slopes, obtained using GenePop, were similar among the three datasets: medians of 1.58E-08, 2.00E-08 and 2.23E-08 for <20%, <15% and <5% missing data respectively (fig. S14). These datasets would therefore have similar neighbourhood size estimates. However, we note that the regression with the 5% missing data threshold had confidence intervals overlapping 0. This is perhaps not surprising given the weak positive relationship at less stringent filtering levels.

N_e estimates obtained using NeEstimator were similar among < 20% and < 15% missingness datasets but were substantially lower when filtering for < 5% missing data (table S8). This is consistent with previous empirical studies investigating the effect of missing data on N_e estimation, whereby higher levels of missing data result in a downward bias in N_e estimates (*56,57*). However, this dataset consists of only 122 SNPs and at least a few thousand SNPs are recommended for accurate N_e estimation, especially when true N_e is large (*117*).

Isolation by distance analyses and error propagation

Census counts, population size and population density

The number of *S. pistillata* and *P. verrucosa* coral colonies at each sampling site was obtained using photogrammetry data, where all colonies of the target species within the photogrammetry plots where identified and digitized. Each sampling site consisted of four 12 x 6 meters plots, summing up to a surface area of 288 m^2 per site. In *S. pistillata,* sympatric cryptic species that are not distinguishable on photogrammetry images exist at these sites *(58)* and we therefore needed to scale census numbers by the relative abundance of *S. pistillata* Taxon1 at each site (data available from (*47*)). To account for the double uncertainties per reef (census density of *S. pistillata* morphospecies and relative proportion of Taxon 1), we fit an intercept only model with a Poisson distribution to describe raw counts from photogrammetry and an intercept only model with a binomial distribution to describe Taxon1 relative abundances. We multiplied these two probably distributions to propagate reef-level uncertainty for Taxon1 census population size and obtain a probability distribution for *S. pistillata* Taxon1 N_c. We note that although cryptic *S. pistillata* species could differ in proportion by depth, our sample sizes by depth for each site were small and our procedures to estimate N_c could not account for this source of variation. For *P.*

verrucosa, we fit an intercept only model with a Poisson distribution to describe raw counts from photogrammetry to obtain a probability distribution for N_c.

Next, we modelled census density (D_c) distributions. For *S. pistillata*, we used a 2-dimensional IbD model and therefore divided the N_c distribution by the area of the sampling site (4 x 72 = 288 m^2) to obtain the D_c distribution. For *P. verrucosa,* we used a 1-dimensional IbD model and therefore needed to use a linear density. We viewed each sampling site as a belt transect of 0.5 m x (12 x 48) m (quasi linear) and divided the N_c distribution by 12 x 48 (= 576) to obtain a D_c distribution. We additionally multiplied the D_c distribution by 290000/2000000, which is the ratio of the coral-covered length on the GBR (obtained via Coral Allen Atlas). Scripts with detailed procedures for all estimated distributions are available on GitHub (https://github.com/zoemeziere/coral_connectivity).

Effective population size and population density

To estimate N_e, we used the linkage disequilibrium method implemented in NeEstimator (*55*), with a non-random mating model. N_e is appropriately estimated at the geographic scale of the neighbourhood size, so we estimated N_e at the reef-level for *S. pistillata.* For *P. verrucosa*, we estimated N_e at different spatial scales and the smallest scale at which we could obtain non-infinite values was at an overlapping regional level with two populations: all individuals from Southern and Central populations as one population (Flinders, Heron, Lady Musgrave, Pelorus, Davies, Little Broadhurst, Chicken) and all individuals from Central and Northern populations as a second population (Pelorus, Davies, Little Broadhurst, Chicken, Moore, Lizard, Masig, Aukane, Dungeness). Because rare alleles can bias estimates of N_e, NeEstimator calculates several estimates of N_e after excluding alleles with frequencies less than critical values Pcrit. As recommended by *(117)*, we chose Pcrit depending on the number of samples in the population (S): Pcrit = 0.01 if S > 100, Pcrit = 0.02 if S > 35, and 1/2S < Pcrit < 1/S if S < 25. Then, we corrected for low levels of linkage that could downwardly bias N_e estimates (*118*) using equation (1):

$$N_e = \frac{\widehat{N_e}}{(0.098+0.219 \times \ln(number\ of\ chromosomes))} \qquad (1)$$

The karyotypes of both *S. pistillata* and *P. verrucosa* are not documented, but many Scleractinian coral species have 2n= 28 chromosomes (*119*). NeEstimator returns median N_e estimates and 95% jackknife confidence intervals but not a full distribution. To allow error propagation, we

fitted a gamma distribution to the N_e estimates for each population. A gamma distribution is adequate because we know that N_e is a non-zero integer that can approach infinity. For *S. pistillata*, we used a 2D IbD model and therefore need to divide the N_e distributions by surface areas. We estimated reef-level surface areas using the Coral Allen Atlas (https://allencoralatlas.org/), which reports an estimated surface area for multiple benthic categories. For each reef, we created a polygon between the sampling sites and used the surface area of the "Coral/Algae" benthic category. We divided population-level N_e distributions by the corresponding surface area to obtain population-level effective density (D_e) distributions. For *P. verrucosa*, we used a 1D IbD model and therefore divided the N_e distributions by the length of the area considered, for each population, to obtain linear D_e distributions. The lengths between the southernmost and northern most sampling sites for each population were calculated using the 'sf' R package.

Sigma and neighbourhood size (NS)

For both datasets, we used the mean and 95% confidence interval values for the IbD slope (b) obtained in GenePop to parametrise a log-normal probability distribution. A log-normal distribution is adequate for the regression slope because the confidence intervals obtained using GenePop are asymmetric. Using credible intervals instead of confidence intervals would be more adequate to fit a probability distribution, but GenePop does not provide them. The probability distributions for the slope and for D_c and D_e were then resampled to obtain distributions for sigma, using equations (1) (for *S. pistillata*) and (2) (for *P. verrucosa*), and for the neighbourhood sizes (*NS*), using equations (3) (for *S. pistillata*) and (4) (for *P. verrucosa*):

$$\sigma = \sqrt{\frac{1}{4D\mathrm{b}\pi}} \qquad (1)$$

$$\sigma = \sqrt{\frac{1}{4D\mathrm{b}}} \qquad (2)$$

$$NS = 4D\pi\,\sigma^2 \qquad (3)$$

$$NS = 4D\sigma^2 \qquad (4)$$

Conversion of δaδi estimates into biologically relevant units

To transform the δaδi output estimates, that are scaled by $2N_{ref}$ generations, we first calculated the total sequence length (L) for each population pair using equation (2):

$$L = \frac{SNP_D \times \text{Number of RAD tags} \times \text{lengh of RAD tags}}{SNP_O} \qquad (2)$$

SNP_D is the number of SNPs retained for the δaδi analyses after projection and SNP_O is the number of SNPs originally detected.

Then, to convert δaδi estimates (θ, Nu_1, Nu_2, M_{12}, M_{21} and T) to physical units (N_{ref}, Ne_1, Ne_2, m_{12}, m_{21}, N_em_{12}, N_em_{21} and t) we used $\mu = 1.2 \times 10^{-8}$ (*24*) and equations (3), (4), (5), (6) and (7):

Ancestral effective population size: $N_{ref} = \frac{\theta}{4 \times \mu \times L}$ (3)

Population sizes: $\nu_1 = Nu_1 \times N_{ref}$ and $\nu_2 = Nu_2 \times N_{ref}$ (4)

Migration rates: $m_{12} = \frac{M12}{2 \times Nref}$ and $m_{21} = \frac{M21}{2 \times Nref}$ (5)

Gene flow: $N_em_{12} = m_{12} \times Ne_1$ and $N_em_{21} = m_{21} \times N_{e2}$ (6)

Divergence times: $t = T \times N_{ref} \times 2 \times 3$ (7)

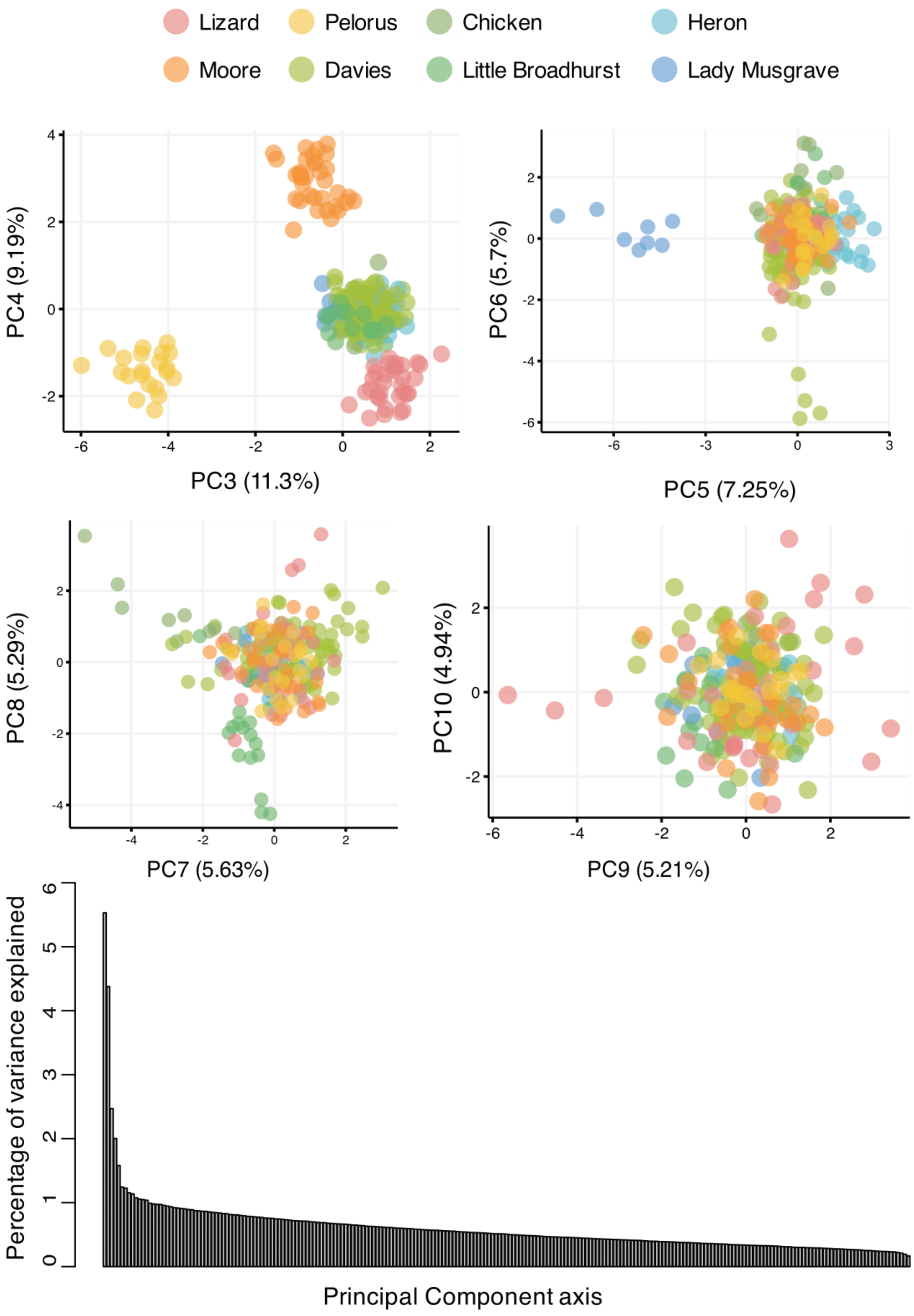

Fig. S1. Additional axes of Principal Component Analysis showing population structure in *Stylophora pistillata*. The dataset includes 232 samples represented by 4,527 reduced representation SNPs. The first 5 axes explained most of the total genetic variance and show reef-level population structure.

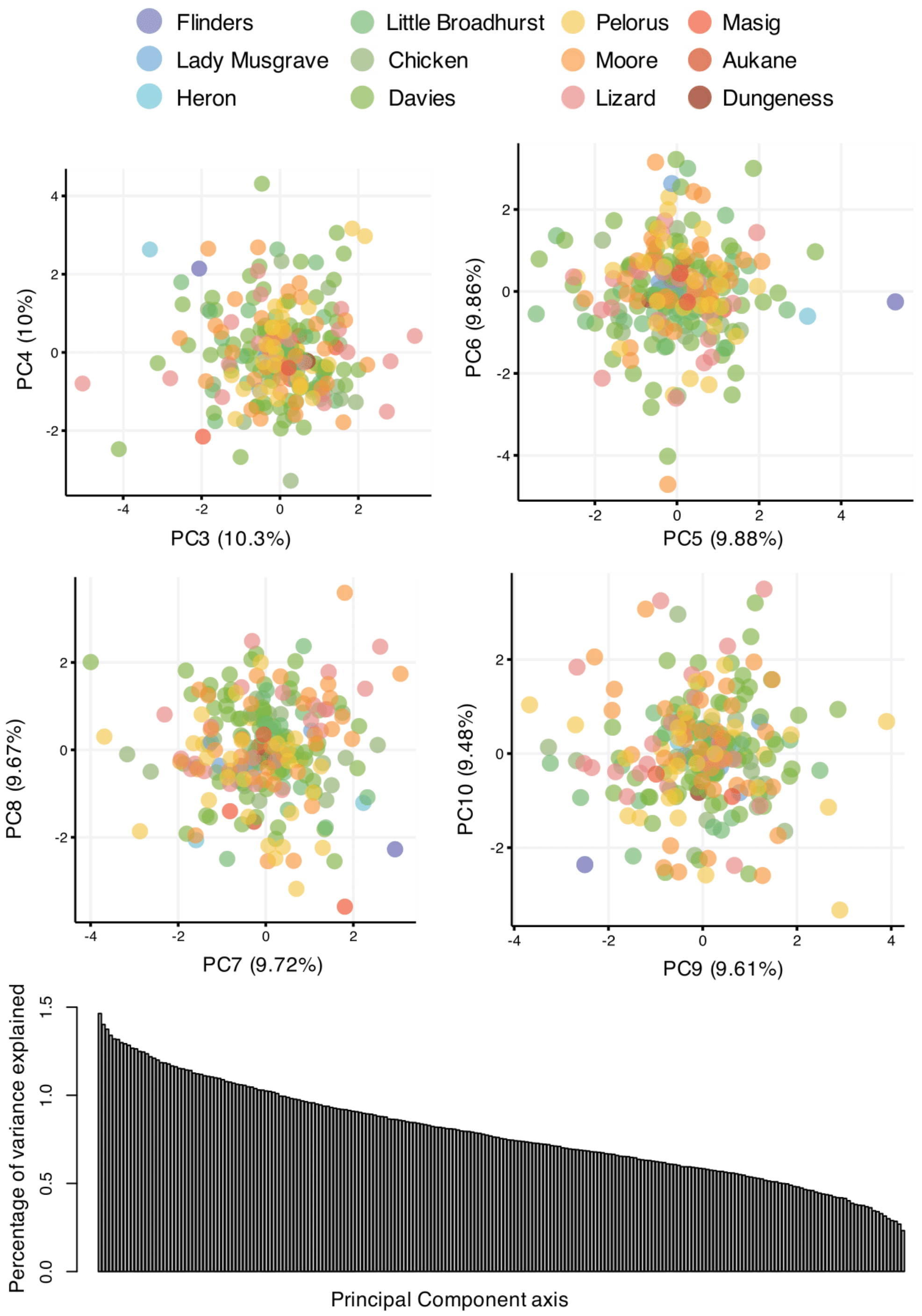

Fig. S2. Additional axes of Principal Component Analysis showing population structure in *Pocillopora verrucosa*. The dataset includes 224 samples represented by 8,597 reduced representation SNPs. All axes explain a small percentage of the total genetic variance and reflect lack of population structure.

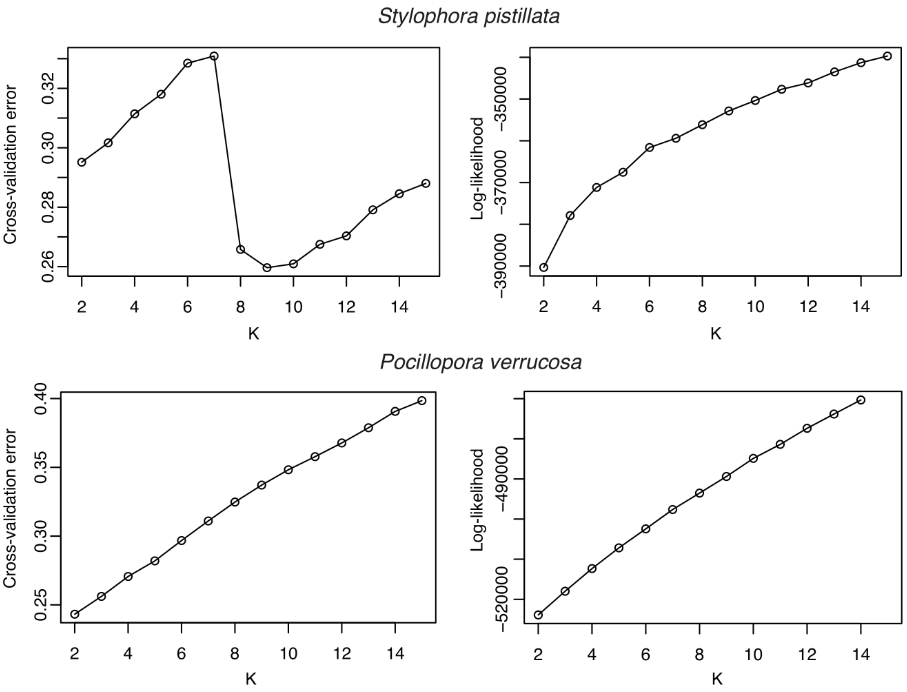

Fig. S3. Cross validation errors and log-likelihood from ADMIXURE runs for both species. ADMIXTURE was run with K=2 to K=15. Results for *Stylophora pistillata* are on the top panels, and results for *Pocillopora verrucosa* are on the bottom panels.

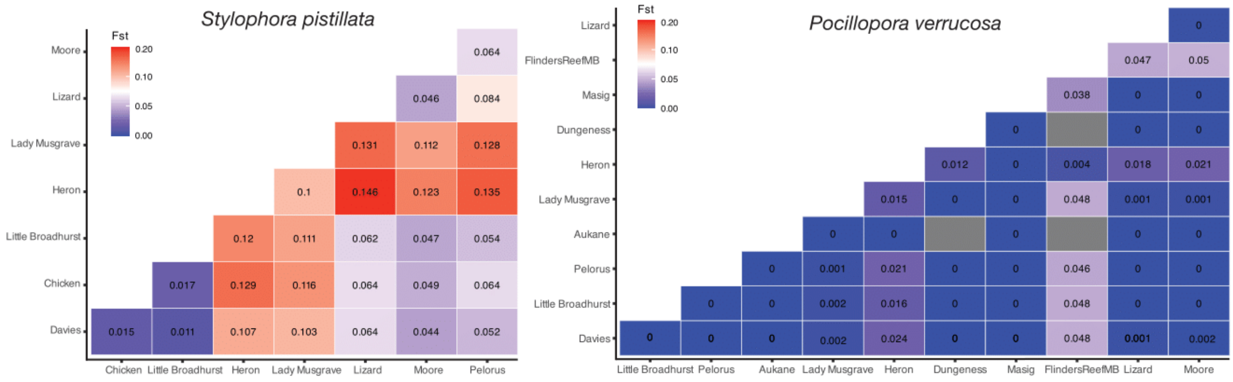

Fig. S4. Higher genetic differentiation between reef-defined populations in *Stylophora pistillata* compared to *Pocillopora verrucosa*. Pairwise population F_{ST} is much higher for all population pairs in *Stylophora pistillata* (left panel) compared to *Pocillopora verrucosa* (right panel).

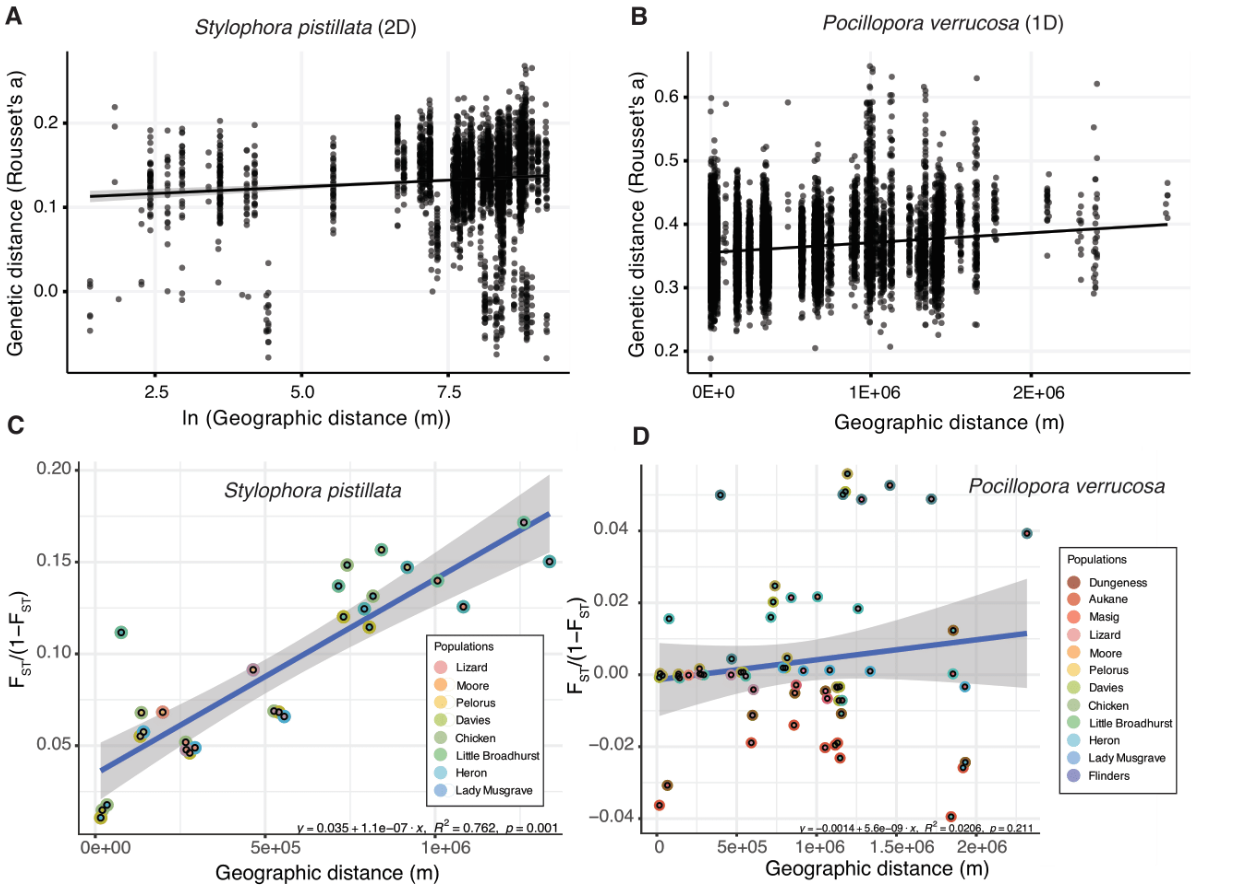

Fig. S5. Individual and population-based isolation by distance (IbD) regressions for *Stylophora pistillata* and *Pocillopora verrucosa*. (A) Individual-based IbD regression for *Stylophora pistillata* using Rousset's a as the genetic distance and a 2-dimentional model for individual pairs separated by less than 1,000 meters; (B) individual-based IbD regression for *Pocillopora verrucosa* using Rousset's a as the genetic distance and a 1-dimentional model for all individual pairs; (C) population-based using $F_{ST}/(1-F_{ST})$ as the genetic distance for all *Stylophora pistillata* populations; (D) population-based using $F_{ST}/(1-F_{ST})$ as the genetic distance for all *Pocillopora verrucosa* populations.

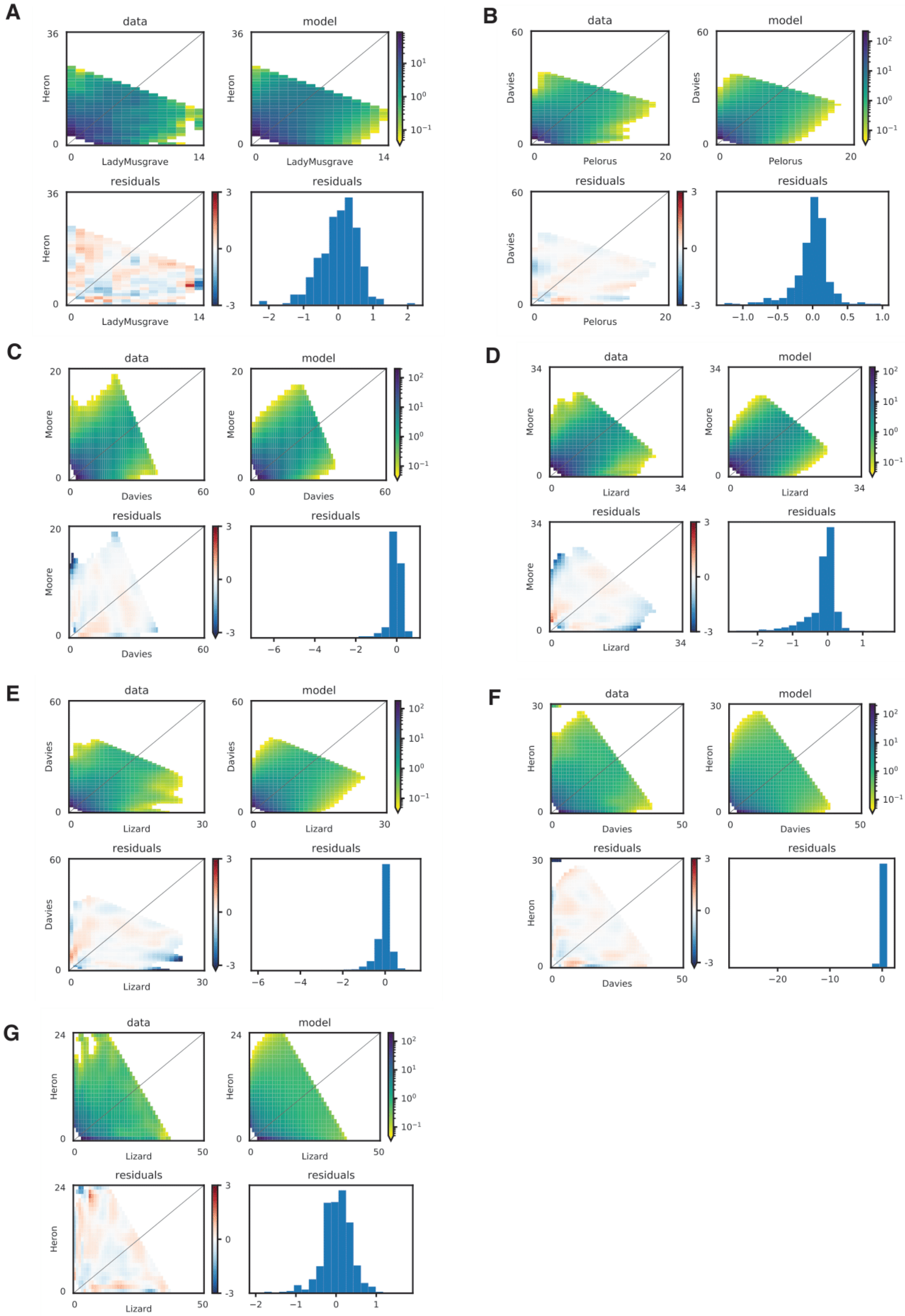

Fig. S6. Data site frequency spectra, simulated site frequency spectra and the standardised residuals between *Stylophora pistillata* population pairs across the Great Barrier Reef. Simulated data was obtained using δaδi with a model of asymmetrical migration between (A) Heron and Lady Musgrave populations; (B) Davies and Pelorus populations; (C) Moore and Davies populations; (D) Lizard and Moore population; (E) Davies and Lizard populations; (F) Heron and Davies populations; (G) Heron and Lizard populations.

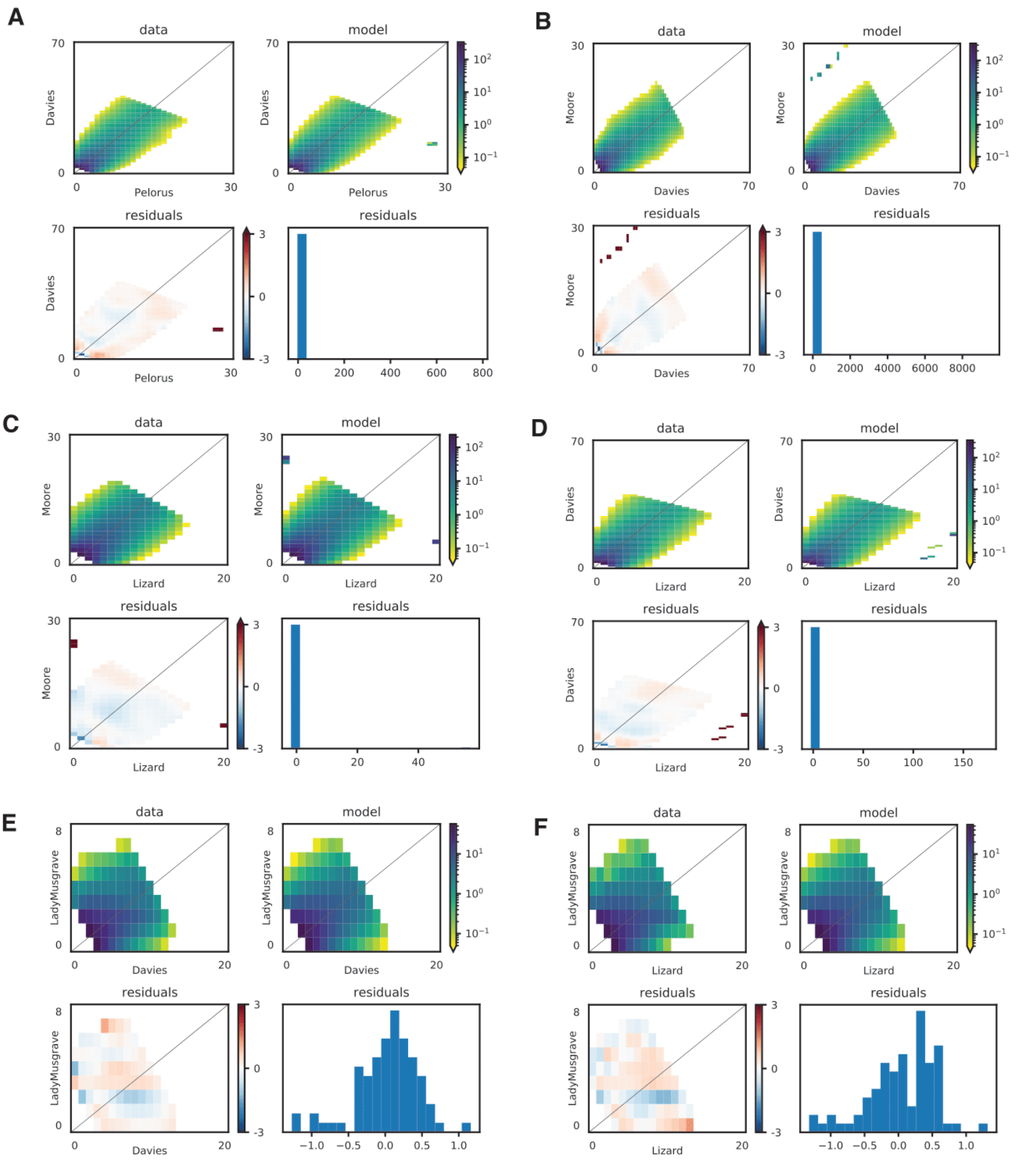

Fig. S7. Data site frequency spectra, simulated site frequency spectra and the standardised residuals between *Pocillopora verrucosa* population pairs across the Great Barrier Reef. Simulated data was obtained using δaδi with a model of asymmetrical migration between (A) Pelorus and Davies populations; (B) Moore and Davies populations; (C) Lizard and Moore populations; (D) Lizard and Davies populations; (E) Davies and Lady Musgrave population; (F) Lizard and Lady Musgrave populations.

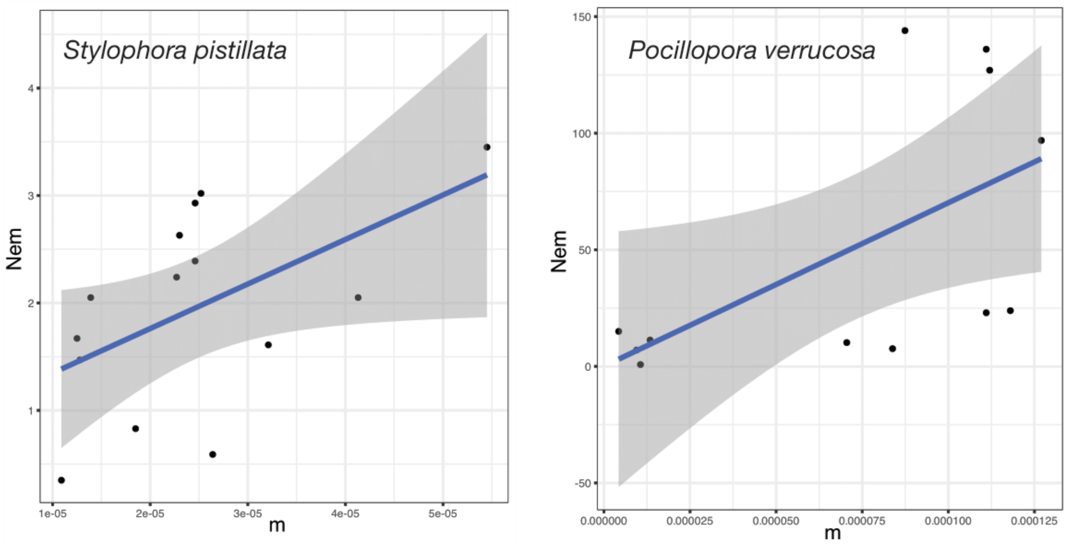

Fig. S8. Linear regressions between the migration rate (m) and gene flow (N_em) estimates between *Stylophora pistillata* and *Pocillopora verrucosa* population pairs. Estimates were obtained using demographic modelling in *δaδi* with an isolation with asymmetrical migration model.

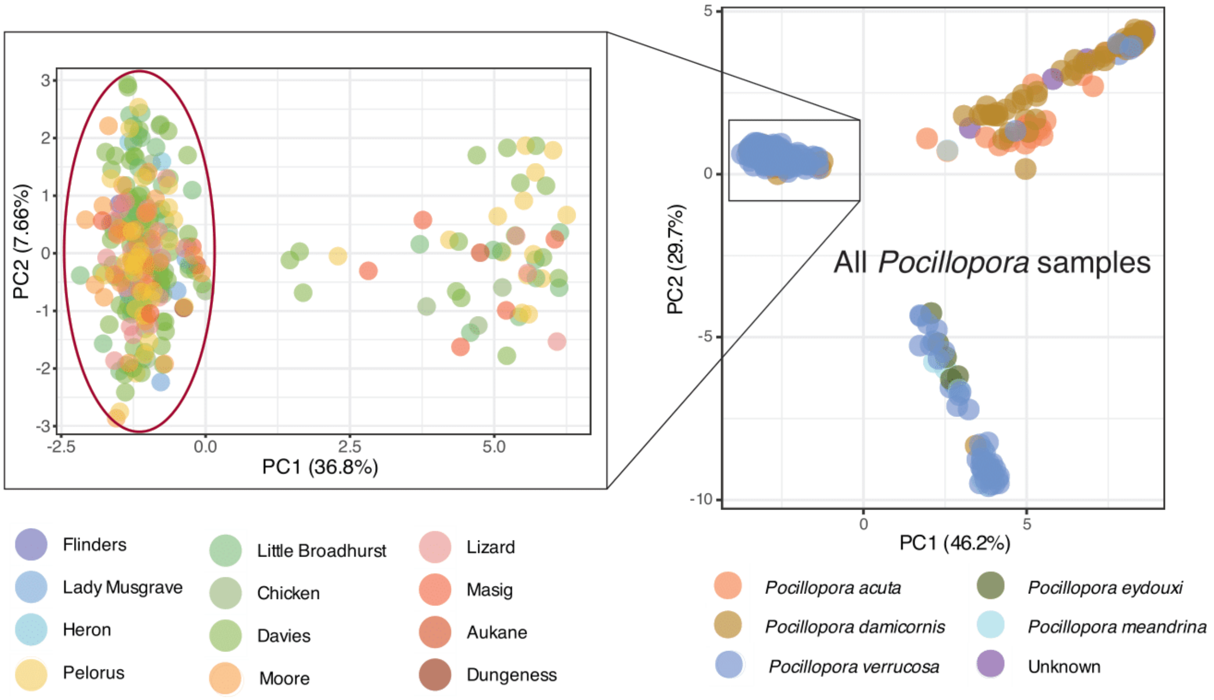

Fig. S9. Population structure among Pocillopora samples identified as different morphospecies. Main panel (right) shows a Principal Component Analysis of 423 *Pocillopora* samples represented by 5,793 reduced representation SNPs. Inset (left) shows Principal Component Analysis of 275 individuals represented by the same 11,189 SNPs, clustered into two

sympatric genetic groups. In this study, we called "*Pocillopora verrucosa*" the cohesive genetic cluster circled in red (224 individuals).

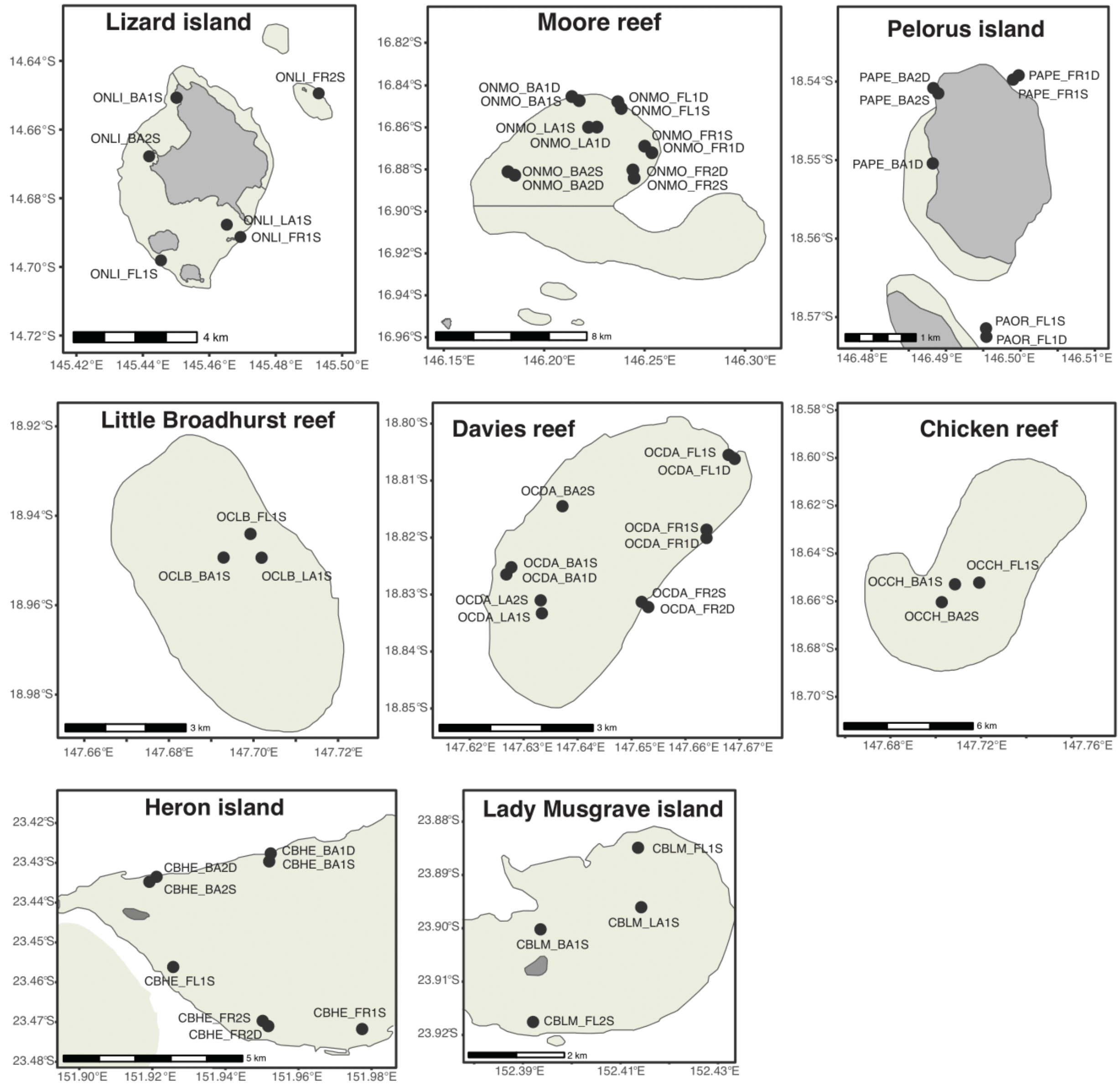

Fig. S10. Maps of all the sampled reefs with more than one sampling site. Grey areas represent islands and beige areas represent reefs. Black dotes are the sampling sites.

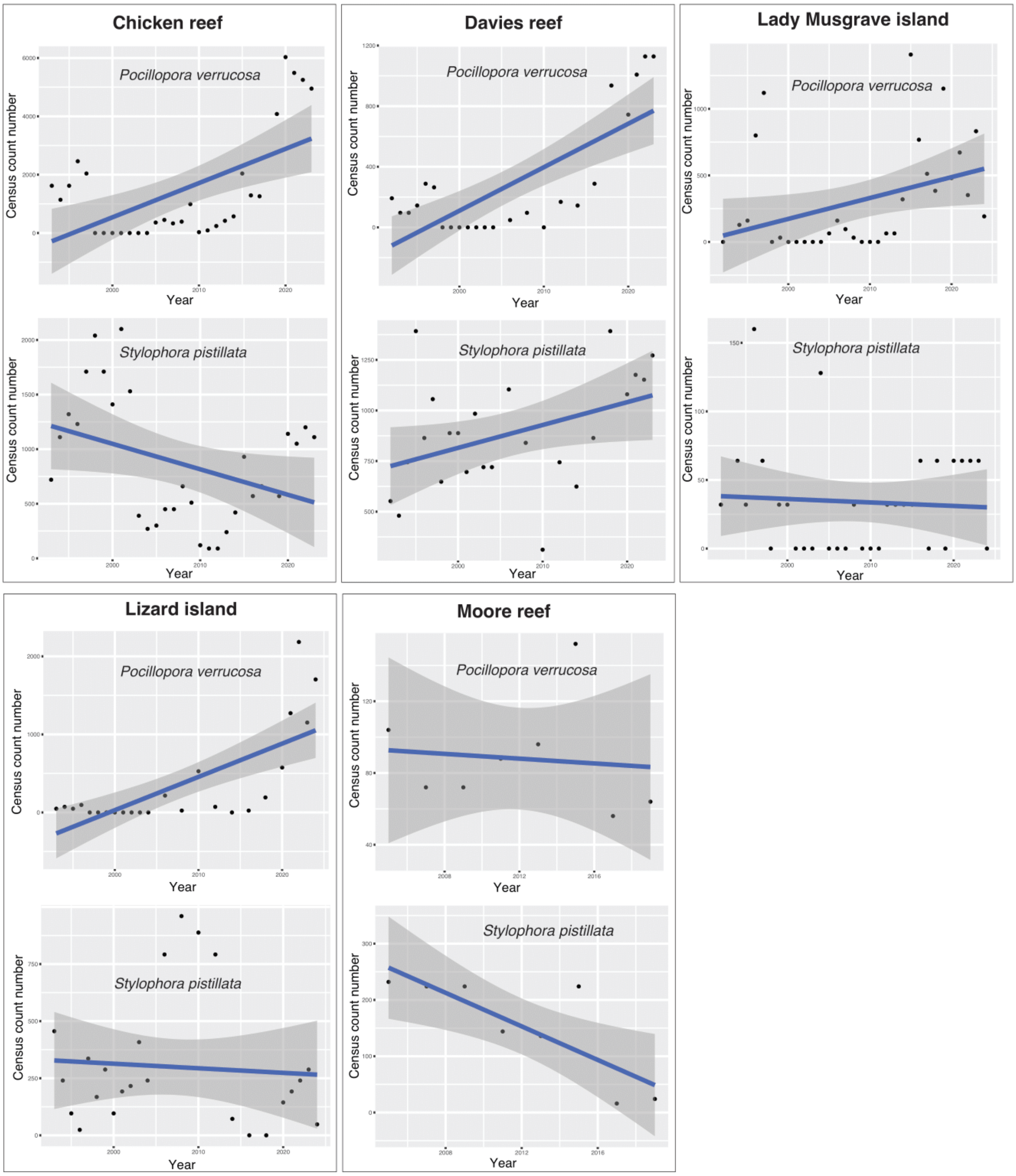

Fig. S11. Linear regressions of census count numbers of *Pocillopora verrucosa* and *Stylophora pistillata* coral colonies between years 1992 and 2024 at sampling locations. This data was generated by the AIMS Long-Term Monitoring Program.

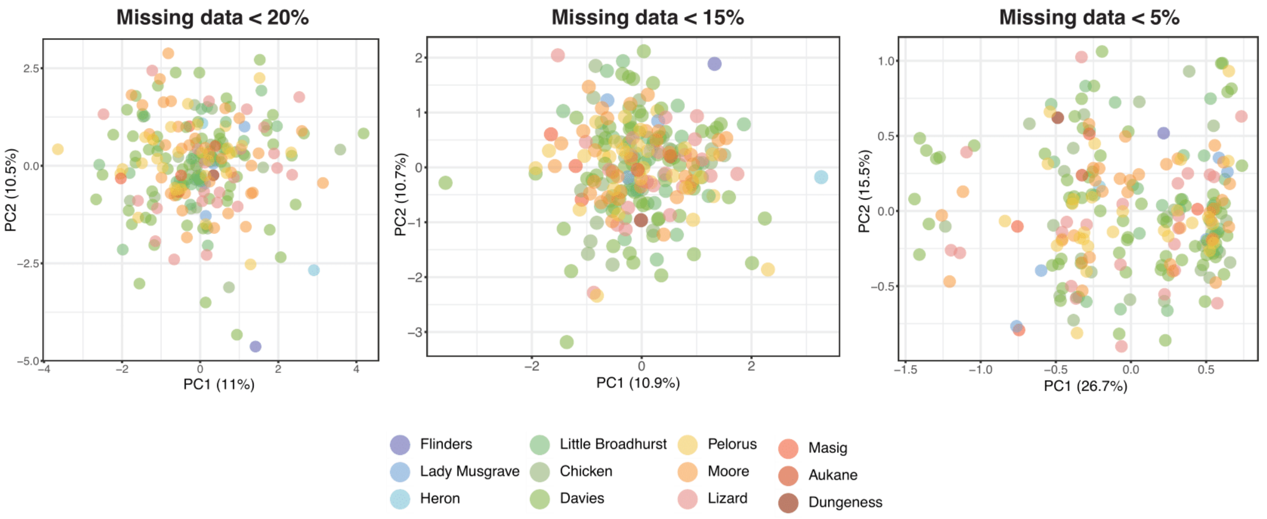

Fig. S12. Principal Component Analyses reveal no population structure in *Pocillopora verrucosa*, regardless of the amount of missing data. From left to right, PCA plots showing population structure with datasets ranging from < 5% to < 20% missing data.

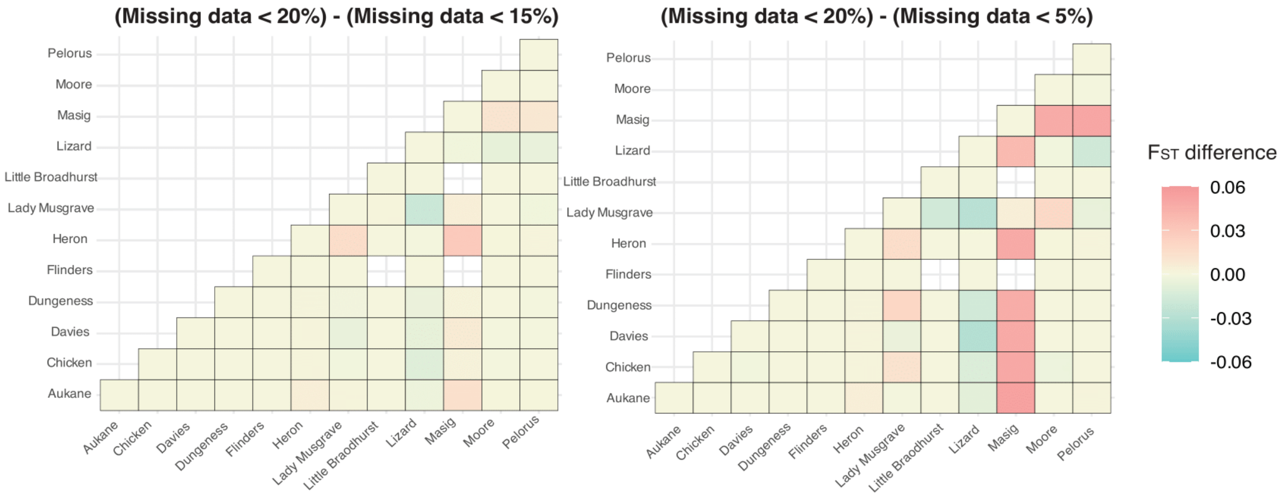

Fig. S13. Differences between pairwise F_{ST} values among *Pocillopora verrucosa* populations, between datasets filtered with different missing data thresholds. Left panel: pairwise F_{ST} value differences between the dataset with < 20% missing data and the dataset with < 15% missing data. Right panel: pairwise F_{ST} value differences between the dataset with < 20% missing data and the dataset with < 5% missing data. Positive values reflect higher F_{ST} in the dataset with more missing data. Negative values reflect lower F_{ST} in the dataset with more missing data.

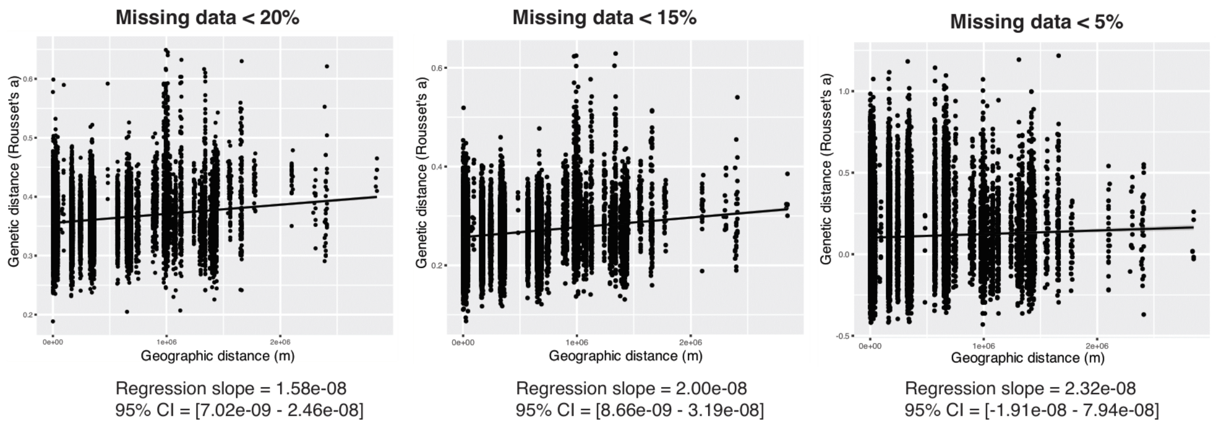

Fig. S14. Isolation by distance regressions have similar slopes among datasets with different levels of missing data. From left to right: Isolation by distance performed in GenePop with datasets ranging from < 5% to < 20% missing data. Median regression slopes as well as 95% confidence intervals are reported above each panel.

Table S1. Sampling sites coordinates and number of *Stylophora pistillata* and *Pocillopora verrucosa* coral colonies sampled at each site. Latitude and longitude coordinates are provided in the WGS84 coordinate system.

Reef	Site	Depth (m)	Latitude	Longitude	*S. pistillata* sampled	*P. verrucosa* sampled
Heron	CBHE_BA1D	12	-23.428	151.952	1	1
Heron	CBHE_FR2D	12	-23.471	151.951	5	0
Heron	CBHE_FR2S	5	-23.470	151.951	1	0
Heron	CBHE_FL1S	5	-23.456	151.926	6	0
Heron	CBHE_BA1S	5	-23.428	151.952	2	0
Heron	CBHE_BA2D	12	-23.434	151.920	4	0
Heron	CBHE_BA2S	5	-23.434	151.921	3	1
Lady Musgrave	CBLM_BA1S	5	-23.900	152.394	0	3
Lady Musgrave	CBLM_FL1S	5	-23.885	152.414	0	2
Lady Musgrave	CBLM_LA1S	5	-23.896	152.414	1	0
Lady Musgrave	CBLM_FL2S	5	-23.917	152.392	7	0
Flinders	FLI_SB1D	10	-26.970	153.480	0	1
Chicken	OCCH_BA1S	5	-18.653	147.708	9	13
Chicken	OCCH_BA2S	5	-18.660	147.703	4	10
Chicken	OCCH_FL1S	5	-18.652	147.718	0	3
Davies	OCDA_BA1D	13	-18.826	147.627	4	6
Davies	OCDA_BA1S	5	-18.826	147.627	8	8
Davies	OCDA_BA2S	5	-18.814	147.637	9	6
Davies	OCDA_FL1D	13	-18.806	147.669	8	7
Davies	OCDA_FL1S	5	-18.806	147.669	11	5
Davies	OCDA_FR1D	13	-18.820	147.664	7	4
Davies	OCDA_FR1S	5	-18.819	147.664	4	10
Davies	OCDA_FR2D	13	-18.832	147.652	7	6
Davies	OCDA_FR2S	5	-18.832	147.652	7	10
Davies	OCDA_LA1S	5	-18.833	147.633	12	8
Davies	OCDA_LA2S	5	-18.831	147.633	4	5
Little Broadhurst	OCLB_BA1S	5	-18.949	147.693	8	8
Little Broadhurst	OCLB_FL1S	5	-18.944	147.699	7	10
Little Broadhurst	OCLB_LA1S	5	-18.949	147.702	6	4
Lizard	ONLI_BA1S	7	-14.651	145.450	3	3
Lizard	ONLI_BA2S	5	-14.668	145.441	6	5
Lizard	ONLI_FL1S	4	-14.698	145.445	3	5
Lizard	ONLI_FR1S	5	-14.691	145.469	8	2
Lizard	ONLI_FR2S	5	-14.649	145.493	7	8

Lizard	ONLI_LA1S	7	-14.649	145.493	5	0
Moore	ONMO_BA1D	12	-16.847	146.217	5	5
Moore	ONMO_BA1S	5	-16.847	146.217	0	2
Moore	ONMO_BA2D	10	-16.882	146.184	5	3
Moore	ONMO_BA2S	5	-16.882	146.184	1	4
Moore	ONMO_FL1D	12	-16.848	146.237	0	4
Moore	ONMO_FL1S	5	-16.848	146.237	0	1
Moore	ONMO_FR1S	5	-16.872	146.254	1	0
Moore	ONMO_FR1D	12	-16.872	146.254	4	2
Moore	ONMO_FR2D	13	-16.883	146.245	5	3
Moore	ONMO_FR2S	5	-16.883	146.245	4	1
Moore	ONMO_LA1D	10	-16.860	146.226	5	5
Moore	ONMO_LA1S	5	-16.860	146.226	4	4
Pelorus	PAPE_FL1D	12	-18.571	146.495	4	2
Pelorus	PAPE_FL1S	5	-18.572	146.495	0	1
Pelorus	PAPE_BA1D	13	-18.550	146.489	3	0
Pelorus	PAPE_BA2D	12	-18.541	146.489	1	3
Pelorus	PAPE_BA2S	3	-18.541	146.489	3	13
Pelorus	PAPE_FR1D	13	-18.539	146.500	10	4
Pelorus	PAPE_FR1S	5	-18.539	146.500	0	7
Aukane	TSAU_FR2D	14	-9.884	143.407	0	1
Dungeness	TSDU_FR2S	5	-10.037	142.926	0	1
Masig	TSMA_BA1S	4	-9.749	143.400	0	1
Masig	TSMA_FR2S	4	-9.748	143.458	0	2
Masig	TSMA_LA1S	5	-9.734	143.435	0	1

Table S2. Details all numbers of *Stylophora pistillata* and *Pocillopora verrucosa* coral colonies sampled. We report the number of sites sampled at each reef and the total number of coral colonies sampled at each reef.

Reef	Number Sites	*S. pistillata*	*P. verrucosa*
Heron	7	22	2
Lady Musgrave	4	8	5
Flinders	1	0	1
Chicken	3	13	26
Davies	11	81	69
Little Broadhurst	3	21	22
Lizard	6	32	23
Moore	12	34	34
Pelorus	7	21	30
Aukane	1	0	1
Dungeness	1	0	1
Masig	3	0	4

Table S3. Demographic modelling estimated parameters for focal *Stylophora pistillata* populations. We report δaδi scaled estimated parameters: theta (Θ), migration rates (Nu_1, Nu_2), gene flow rates (M_{12}, M_{21}) and divergence time (T) and their standard deviations obtained using the Fisher Information Matrix. LM = Lady Musgrave.

		δaδi estimated parameters						δaδi estimated parameters standard deviations					
**Populations**	**Projection**	**Θ**	**Nu_1**	**Nu_2**	**T**	**M_{12}**	**M_{21}**	**Θ_SD**	**Nu_1_SD**	**Nu_2_SD**	**T_SD**	**M_{12}_SD**	**M_{21}_SD**
**Heron-LM**	36-14	227	1.29	1.44	2.44	2.19	1.24	19.2	0.24	0.23	0.29	0.32	0.25
**Pelorus-Davies**	20-60	252	3.34	1.38	1.79	2.96	1.81	66.6	0.83	0.54	0.82	2.31	0.49
**Moore-Davies**	20-60	335	2.21	2.12	1.04	2.65	2.48	25.9	0.41	0.33	0.17	0.53	0.53
**Lizard-Moore**	34-34	386	1.38	3.21	1.06	4.99	1.27	35.6	0.48	0.61	0.22	1.62	0.46
**Lizard-Davies**	30-60	263	2.44	2.88	1.65	1.95	1.02	22.5	0.32	0.35	0.23	0.24	0.18
**Davies-Heron**	50-30	250	3.69	0.83	1.84	1.21	1.41	11.9	0.33	0.05	0.08	0.14	0.06
**Lizard-Heron**	50-24	201	3.87	0.95	1.64	0.86	0.75	7.86	0.34	0.16	0.07	0.03	0.17

Table S4. Demographic modelling estimated parameters for focal *Pocillopora verrucosa* populations. We report δaδi scaled estimated parameters: theta (Θ), migration rates (Nu_1, Nu_2), gene flow rates (M_{12}, M_{21}) and divergence time (T) and their standard deviations obtained using the Fisher Information Matrix. LM = Lady Musgrave.

		δaδi estimated parameters						δaδi estimated parameters standard deviations					
**Populations**	**Projection**	**Θ**	**Nu_1**	**Nu_2**	**T**	**M_{12}**	**M_{21}**	**Θ_SD**	**Nu_1_SD**	**Nu_2_SD**	**T_SD**	**M_{12}_SD**	**M_{21}_SD**
**Pelorus-Davies**	30-70	400	12.7	2.5	1.2	20.0	18.6	1.2	1.3E-03	7.5E-05	3.2E-05	2.7E-03	1.1E-03
**Moore-Davies**	30-70	384	2.6	9.7	1.2	18.6	20.0	7.9	0.014	0.074	0.021	0.53	0.17
**Lizard-Moore**	20-30	353	6.4	14.4	1.4	0.24	20.0	8.5	0.18	0.37	0.027	6.3E-03	0.51
**Lizard-Davies**	20-70	403	4.0	13.6	1.1	7.6	20.0	25.1	1.8	8.4	0.12	8.4	5.3
**Davies-LM**	20-8	49	10.3	2.0	5.0	1.4	10.2	31.6	2.0	6.2	4.0	1.5	9.4
**Lizard-LM**	20-8	80	7.1	0.76	2.57	3.2	20.0	55.2	4.1	1.4	2.6	4.6	39.3

Table S5. **Estimates of contemporary effective population sizes and estimates of surface area or length used to obtain effective population densities**. Area is reported in m^2 for *Stylophora pistillata* (2D IbD model) and length is reported in meters for *P. verrucosa* (1D IbD model). Pcrit is the critical value used to exclude low-frequency alleles. $\widehat{N_e}$ is the uncorrected effective population size obtained using NeEstimator, with the 95% confidence interval in parenthesis. Coral-covered surface areas and lengths were obtained using the Coral Allen Atlas (Coral/Algae category).

Species	Population	Pcrit	$\widehat{N_e}$	Area / Length
S. pistillata	Lizard	0.02	251.1 (239.1 - 264.3)	170,000
	Moore	0.02	594.7 (534.6 - 669.6)	350,000
	Pelorus	0.05	114.3 (107.4 - 122.2)	40,000
	Davies	0.02	964 (904.4 - 1031.7)	390,000
	Chicken	0.05	131.4 (117.4 - 149.2)	50,000
	Little Broadhurst	0.05	854 (605.5 - 1443)	10,000
	Heron	0.05	423.6 (332.2 - 582.5)	620,000
	Lady Musgrave	0.05	51.2 (45.9 - 57.8)	1,080,000
P. verrucosa	South	0.01	30,756.8 (20,567.7 – 60,919.5)	1,200,000
	North	0.02	43,158.4 (28,553.4 – 88,273.7)	1,125,000

Table S6. ***Stylophora pistillata* and *Pocillopora verrucosa* census count numbers at sampling sites**. Census count data was obtained from high resolution photogrammetry data. *S. pistillata* Taxon1 abundance relative to other *S. pistillata* cryptic taxa at each sampling site is also reported.

Site	***S. pistillata*** **census count**	***S. pistillata*** **Taxon1 abundance**	***P. verrucosa*** **census count**
CBHE_BA1D	2	0.5	1
CBHE_BA1S	1	0.28	1
CBHE_BA2D	13	0.8	0
CBHE_BA2S	5	0.6	0
CBHE_FL1S	12	0.45	0
CBHE_FR1S	31	0.048	0
CBHE_FR2D	14	1	0
CBHE_FR2S	40	0.2	1
CBLM_FL1S	8	0.54	6
CBLM_FL2S	99	0.5	4
CBLM_LA1S	2	1	0
CBLM_BA1S	0	0	10
CBLM_BA2S	0	0	2
OCCH_BA1S	18	0.5	25

OCCH_FL1S	52	1	33
OCCH_BA2S	0	0	19
OCDA_BA1D	9	0.89	11
OCDA_BA1S	22	1	21
OCDA_BA2D	0	0	6
OCDA_BA2S	30	0.92	24
OCDA_FL1D	34	0.875	18
OCDA_FL1S	74	0.4	28
OCDA_FR1D	83	1	30
OCDA_FR1S	78	0.78	69
OCDA_FR2D	17	1	49
OCDA_FR2S	17	1	56
OCLB_BA1S	50	1	19
OCLB_FL1S	20	1	22
OCLB_LA1S	6	1	1
ONLI_BA1D	0	0	5
ONLI_BA1S	12	1	16
ONLI_BA2S	7	1	11
ONLI_FL1S	2	1	64
ONLI_FR1S	16	1	127
ONLI_FR2S	145	1	59
ONLI_LA1S	14	1	1
ONMO_BA1D	21	1	38
ONMO_BA1S	0	0	75
ONMO_BA2D	0	0	21
ONMO_BA2S	0	0	33
ONMO_FL1D	9	1	23
ONMO_FL1S	20	0.5	61
ONMO_FR1D	21	1	8
ONMO_FR1S	6	1	63
ONMO_FR2D	28	1	2
ONMO_FR2S	16	1	69
ONMO_LA1D	48	0.67	0
ONMO_LA1S	36	1	3
PAPE_FR1D	1	1	1
PAPE_FL12	0	0	1
PAPE_BA2D	0	0	1
PAPE_BA2S	0	0	6
PAPE_FR1D	0	0	1
PAPE_FR1S	0	0	4
TSAU_FR2D	0	0	1
TSMA_FR2S	0	0	2

Table S7. Distances between kin individuals support short larval dispersal in *Stylophora pistillata* on the Great Barrier Reef. In the absence of coordinates for every individual coral colony, we estimated maximum distances (in meters) between kin individuals found at the same sampling site. Kin individuals found in the same 6x12m sampling plot are separated by a maximum distance of 10 m and kin individuals found at the same site but with no information on the sampling plot were separated by a maximum distance of 100 m.

Kinship	Reef	Distance (m)
**Parent - offspring**	Lady Musgrave	3,263
	Lady Musgrave	<10
	Pelorus	<100
	Pelorus	<100
	Pelorus	<100
	Pelorus	<100
	Pelorus	3,606
**Full siblings**	Pelorus	<100
**Half siblings**	Lady Musgrave	<10
	Moore	<10
	Lizard	<100
	Lizard	<100
	Lizard	2,686
	Pelorus	<100
	Davies	<100
	Davies	<100
	Davies	<10
	Davies	15
	Davies	15
	Davies	15
	Davies	15
	Davies	<100
	Davies	<100
	Chicken	<10
	Chicken	<100
	Chicken	<100
	Chicken	<100
	Little Broadhurst	<10

Table S8. Estimates of effective population sizes for *Pocillopora verrucosa*, with different levels of missing data allowed in the dataset. Estimates were obtained using NeEstimator, and levels of missing data ranged from < 5% to < 20%. N_e was estimated for the Southern/ Central GBR reefs and the Northern/ Central GBR reefs. Median estimate as well as low and high 95% confidence intervals are reported.

	N_e South/ Central GBR			N_e North/ Central GBR		
	**Median**	**Low CI**	**High CI**	**Median**	**Low CI**	**High CI**
**< 20%**	43,000	28,000	88,000	31,000	20,000	61,000
**< 15%**	17,000	12,000	29,000	17,000	11,000	39,000
**< 5%**	188	154	238	176	140	232

REFERENCES AND NOTES

1. O. Hoegh-Guldberg, P. J. Mumby, A. J. Hooten, R. S. Steneck, P. Greenfield, E. Gomez, C. D. Harvell, P. F. Sale, A. J. Edwards, K. Caldeira, N. Knowlton, C. M. Eakin, R. Iglesias-Prieto, N. Muthiga, R. H. Bradbury, A. Dubi, M. E. Hatziolos, Coral reefs under rapid climate change and ocean acidification. *Science* **318**, 1737–1742 (2007).

2. K. E. Carpenter, M. Abrar, G. Aeby, R. B. Aronson, S. Banks, A. Bruckner, A. Chiriboga, J. Cortés, J. C. Delbeek, L. DeVantier, G. J. Edgar, A. J. Edwards, D. Fenner, H. M. Guzmán, B. W. Hoeksema, G. Hodgson, O. Johan, W. Y. Licuanan, S. R. Livingstone, E. R. Lovell, J. A. Moore, D. O. Obura, D. Ochavillo, B. A. Polidoro, W. F. Precht, M. C. Quibilan, C. Reboton, Z. T. Richards, A. D. Rogers, J. Sanciangco, A. Sheppard, C. Sheppard, J. Smith, S. Stuart, E. Turak, J. E. N. Veron, C. Wallace, E. Weil, E. Wood, One-third of reef-building corals face elevated extinction risk from climate change and local impacts. *Science* **321**, 560–563 (2008).

3. M. P. Berg, E. T. Kiers, G. Driessen, M. Van Der Heijden, B. W. Kooi, F. Kuenen, M. Liefting, H. A. Verhoef, J. Ellers, Adapt or disperse: Understanding species persistence in a changing world. *Glob. Chang. Biol.* **16**, 587–598 (2010).

4. M. Baguette, S. Blanchet, D. Legrand, V. M. Stevens, C. Turlure, Individual dispersal, landscape connectivity and ecological networks. *Biol. Rev. Camb. Philos. Soc.* **88**, 310–326 (2013).

5. O. Ronce, How does it feel to be like a rolling stone? Ten questions about dispersal evolution. *Annu. Rev. Ecol. Evol. Syst.* **38**, 231–253 (2007).

6. S. Wright, Isolation by distance under diverse systems of mating. *Genetics* **31**, 39–59 (1946).

7. L. B. Slobodkin, Stochastic population models in ecology. *Ecology* **42**, 852–852 (1961).

8. T. J. Kawecki, Demography of source-sink populations and the evolution of ecological niches. *Evol. Ecol.* **9**, 38–44 (1995).

9. M. Slatkin, Gene flow and the geographic structure of natural populations. *Science* **236**, 787–792 (1987).

10. S. Wright, The genetical struture of populations. *Ann. Eugen.* **15**, 323–354 (1949).

11. J. Felsenstein, The theoretical population genetics of variable selection and migration. *Annu. Rev. Genet.* **10**, 253–280 (1976).

12. J. B. S. Haldane, A mathematical theory of natural and artificial selection. (Part VI, Isolation.). *Math. Proc. Camb. Philos. Soc.* **26**, 220–230 (1930).

13. C. L. Morjan, L. H. Rieseberg, How species evolve collectively: Implications of gene flow and selection for the spread of advantageous alleles. *Mol. Ecol.* **13**, 1341–1356 (2004).

14. S. Wright, Isolation by distance. *Genetics* **28**, 114–138 (1943).

15. F. Rousset, Genetic differentiation and estimation of gene flow from *F*-statistics under isolation by distance. *Genetics* **145**, 1219–1228 (1997).

16. Rousset, Genetic differentiation between individuals. *J. Evol. Biol.* **13**, 58–62 (1999).

17. A. De Jode, A. Le Moan, K. Johannesson, R. Faria, S. Stankowski, A. M. Westram, R. K. Butlin, M. Rafajlović, C. Fraïsse, Ten years of demographic modelling of divergence and speciation in the sea. *Evol. Appl.* **16**, 542–559 (2023).

18. J. Hey, Isolation with migration models for more than two populations. *Mol. Biol. Evol.* **27**, 905–920 (2010).

19. L. Excoffier, I. Dupanloup, E. Huerta-Sánchez, V. C. Sousa, M. Foll, Robust demographic inference from genomic and SNP data. *PLOS Genet.* **9**, e1003905 (2013).

20. R. N. Gutenkunst, R. D. Hernandez, S. H. Williamson, C. D. Bustamante, Inferring the joint demographic history of multiple populations from multidimensional SNP frequency data. *PLOS Genet.* **5**, e1000695 (2009).

21. C. Riginos, K. Hock, A. M. Matias, P. J. Mumby, M. J. H. Van Oppen, V. Lukoschek, Asymmetric dispersal is a critical element of concordance between biophysical dispersal

models and spatial genetic structure in Great Barrier Reef corals. *Divers. Distrib.* **25**, 1684–1696 (2019).

22. K. Tsuchiya, Y. Zayasu, Y. Nakajima, N. Arakaki, G. Suzuki, N. Satoh, C. Shinzato, Genomic analysis of a reef-building coral, *Acropora digitifera*, reveals complex population structure and a migration network in the Nansei Islands, Japan. *Mol. Ecol.* **31**, 5270–5284 (2022).

23. M. V. Matz, E. A. Treml, G. V. Aglyamova, L. K. Bay, Potential and limits for rapid genetic adaptation to warming in a Great Barrier Reef coral. *PLOS Genet.* **14**, e1007220 (2018).

24. J. Zhang, Z. T. Richards, A. A. S. Adam, C. X. Chan, C. Shinzato, J. Gilmour, L. Thomas, J. M. Strugnell, D. J. Miller, I. Cooke, Evolutionary responses of a reef-building coral to climate change at the end of the last glacial maximum. *Mol. Biol. Evol.* **39**, msac201 (2022).

25. C. R. Gazulla, P. López-Sendino, A. Antunes, D. Aurelle, I. Montero-Serra, J.-M. Dominici, C. Linares, J. Garrabou, J.-B. Ledoux, Demo-genetic approach for the conservation and restoration of a habitat-forming octocoral: The case of Red Coral, Corallium rubrum, in the Réserve Naturelle de Scandola. *Front. Mar. Sci.* **8**, 633057 (2021).

26. K. D. Gorospe, S. A. Karl, Genetic relatedness does not retain spatial pattern across multiple spatial scales: Dispersal and colonization in the coral, *Pocillopora damicornis*. *Mol. Ecol.* **22**, 3721–3736 (2013).

27. A. Japaud, C. Bouchon, H. Magalon, C. Fauvelot, Geographic distances and ocean currents influence Caribbean Acropora palmata population connectivity in the Lesser Antilles. *Conserv. Genet.* **20**, 447–466 (2019).

28. K. E. Prata, P. Bongaerts, J. M. Dwyer, H. Ishida, S. M. Howitt, J. P. Hereward, E. D. Crandall, C. Riginos, Some reef-building corals only disperse metres per generation. *Proc. Biol. Sci.* **291**, 20231988 (2024).

29. R. Cowen, G. Gawarkiewicz, J. Pineda, S. Thorrold, F. Werner, Population connectivity in marine systems: An overview. *Oceanography* **20**, 14–21 (2007).

30. D. J. Ayre, T. P. Hughes, Genotypic diversity and gene flow in brooding and spawning corals along the great barrier reef, Australia. *Evolution* **54**, 1590–1605 (2000).

31. P. Bongaerts, C. Riginos, R. Brunner, N. Englebert, S. R. Smith, O. Hoegh-Guldberg, Deep reefs are not universal refuges: Reseeding potential varies among coral species. *Sci. Adv.* **3**, e1602373 (2017).

32. L. Thomas, J. N. Underwood, A. A. S. Adam, Z. T. Richards, L. Dugal, K. J. Miller, J. P. Gilmour, Contrasting patterns of genetic connectivity in brooding and spawning corals across a remote atoll system in northwest Australia. *Coral Reefs* **39**, 55–60 (2020).

33. J. N. Underwood, Z. Richards, O. Berry, D. Oades, A. Howard, J. P. Gilmour, Extreme seascape drives local recruitment and genetic divergence in brooding and spawning corals in remote north-west Australia. *Evol. Appl.* **13**, 2404–2421 (2020).

34. R. M. Van Der Ven, H. Heyndericx, M. Kochzius, Differences in genetic diversity and divergence between brooding and broadcast spawning corals across two spatial scales in the Coral Triangle region. *Mar. Biol.* **168**, 17 (2021).

35. M. C. Whitlock, D. E. McCauley, Indirect measures of gene flow and migration: $F_{ST} \neq 1/(4Nm+1)$. *Heredity* **82**, 117–125 (1999).

36. P. B. Marko, M. W. Hart, The complex analytical landscape of gene flow inference. *Trends Ecol. Evol.* **26**, 448–456 (2011).

37. C. Riginos, I. Popovic, Z. Meziere, V. Garcia, I. Byrne, S. M. Howitt, H. Ishida, K. Bairos-Novak, A. Humanes, H. Scharfenstein, T. Richards, E. Briggs, V. Clark, C. Lei, M. Khan, K. E. Prata, Cryptic species and hybridisation in corals: Challenges and opportunities for conservation and restoration. *Peer Community J.* **4**, e106 (2024).

38. R. A. Magris, R. L. Pressey, R. Weeks, N. C. Ban, Integrating connectivity and climate change into marine conservation planning. *Biol. Conserv.* **170**, 207–221 (2014).

39. S. R. Palumbi, Population genetics, demographic connectivity, and the design of marine reserves. *Ecol. Appl.* **13**, 146–158 (2003).

40. IPCC. 2007. Climate Change 2007: Synthesis Report. Contribution of Working Groups I, II and III to the Fourth Assessment Report of the Intergovernmental Panel on Climate Change [Core Writing Team, Pachauri, R. K and Reisinger, A. (eds.)]. IPCC, Geneva, Switzerland, 104 pp.

41. I. B. Baums, A restoration genetics guide for coral reef conservation. *Mol. Ecol.* **17**, 2796–2811 (2008).

42. M. J. H. van Oppen, J. K. Oliver, H. M. Putnam, R. D. Gates, Building coral reef resilience through assisted evolution. *Proc. Natl. Acad. Sci. U.S.A.* **112**, 2307–2313 (2015).

43. K. M. Quigley, L. K. Bay, M. J. H. Oppen, The active spread of adaptive variation for reef resilience. *Ecol. Evol.* **9**, 11122–11135 (2019).

44. M. Hagedorn, C. A. Page, K. L. O'Neil, D. M. Flores, L. Tichy, T. Conn, V. F. Chamberland, C. Lager, N. Zuchowicz, K. Lohr, H. Blackburn, T. Vardi, J. Moore, T. Moore, I. B. Baums, M. J. A. Vermeij, K. L. Marhaver, Assisted gene flow using cryopreserved sperm in critically endangered coral. *Proc. Natl. Acad. Sci. U.S.A.* **118**, e2110559118 (2021).

45. M. C. Urban, G. Bocedi, A. P. Hendry, J.-B. Mihoub, G. Pe'er, A. Singer, J. R. Bridle, L. G. Crozier, L. De Meester, W. Godsoe, A. Gonzalez, J. J. Hellmann, R. D. Holt, A. Huth, K. Johst, C. B. Krug, P. W. Leadley, S. C. F. Palmer, J. H. Pantel, A. Schmitz, P. A. Zollner, J. M. J. Travis, Improving the forecast for biodiversity under climate change. *Science* **353**, aad8466 (2016).

46. Z. Meziere, I. Popovic, K. Prata, I. Ryan, J. Pandolfi, C. Riginos, Exploring coral speciation: Multiple sympatric *Stylophora pistillata* taxa along a divergence continuum on the Great Barrier Reef. *Evol. Appl.* **17**, e13644 (2024).

47. D. H. Alexander, J. Novembre, K. Lange, Fast model-based estimation of ancestry in unrelated individuals. *Genome Res.* **19**, 1655–1664 (2009).

48. F. Rousset, GENEPOP'007: A complete re-implementation of the GENEPOP software for Windows and Linux. *Mol. Ecol. Resour.* **8**, 103–106 (2008).

49. O. R. Jones, J. Wang, COLONY: A program for parentage and sibship inference from multilocus genotype data. *Mol. Ecol. Resour.* **10**, 551–555 (2010).

50. R. S. Waples, What Is *N*e, Anyway? *J. Hered.* **113**, 371–379 (2022).

51. K. L. Korunes, K. Samuk, PIXY: Unbiased estimation of nucleotide diversity and divergence in the presence of missing data. *Mol. Ecol. Resour.* **21**, 1359–1368 (2021).

52. J. Catchen, P. A. Hohenlohe, S. Bassham, A. Amores, W. A. Cresko, Stacks: An analysis tool set for population genomics. *Mol. Ecol.* **22**, 3124–3140 (2013).

53. Z. A. Szpiech, M. Jakobsson, N. A. Rosenberg, ADZE: A rarefaction approach for counting alleles private to combinations of populations. *Bioinformatics* **24**, 2498–2504 (2008).

54. C. Do, R. S. Waples, D. Peel, G. M. Macbeth, B. J. Tillett, J. R. Ovenden, NeEstimator v2: Re-implementation of software for the estimation of contemporary effective population size (N_e) from genetic data. *Mol. Ecol. Resour.* **14**, 209–214 (2014).

55. R. Gargiulo, V. Decroocq, S. C. González-Martínez, I. Paz-Vinas, J. Aury, I. Lesur Kupin, C. Plomion, S. Schmitt, I. Scotti, M. Heuertz, Estimation of contemporary effective population size in plant populations: Limitations of genomic datasets. *Evol. Appl.* **17**, e13691 (2024).

56. F. Marandel, G. Charrier, J. Lamy, S. Le Cam, P. Lorance, V. M. Trenkel, Estimating effective population size using RADseq: Effects of SNP selection and sample size. *Ecol. Evol.* **10**, 1929–1937 (2020).

57. M. L. Pinsky, S. R. Palumbi, S. Andréfouët, S. J. Purkis, Open and closed seascapes: Where does habitat patchiness create populations with high fractions of self-recruitment? *Ecol. Appl.* **22**, 1257–1267 (2012).

58. T. P. Hughes, J. T. Kerry, A. H. Baird, S. R. Connolly, A. Dietzel, C. M. Eakin, S. F. Heron, A. S. Hoey, M. O. Hoogenboom, G. Liu, M. J. McWilliam, R. J. Pears, M. S. Pratchett, W. J. Skirving, J. S. Stella, G. Torda, Global warming transforms coral reef assemblages. *Nature* **556**, 492–496 (2018).

59. AIMS Long-term Monitoring Program. Australian Institute of Marine Science (AIMS). 2015. https://apps.aims.gov.au/metadata/view/a17249ab-5316-4396-bb27-29f2d568f727, accessed 09-Sep-2024.

60. P. L. Harrison, C. C. Wallace “Reproduction, dispersal and recruitment of scleractinian corals” in *Coral Reefs*, Z. Dubinsky Ed. (Elsevier, 1990)25, 133–207.

61. E. A. Treml, P. N. Halpin, Marine population connectivity identifies ecological neighbors for conservation planning in the Coral Triangle. *Conserv. Lett.* **5**, 441–449 (2012).

62. S. Wood, C. B. Paris, A. Ridgwell, E. J. Hendy, Modelling dispersal and connectivity of broadcast spawning corals at the global scale. *Glob. Ecol. Biogeogr.* **23**, 1–11 (2014).

63. A. Saint-Amand, J. Lambrechts, E. Hanert, Biophysical models resolution affects coral connectivity estimates. *Sci. Rep.* **13**, 9414 (2023).

64. M. J. H. Van Oppen, V. Lukoschek, R. Berkelmans, L. M. Peplow, A. M. Jones, A population genetic assessment of coral recovery on highly disturbed reefs of the Keppel Island archipelago in the southern Great Barrier Reef. *PeerJ* **3**, e1092 (2015).

65. V. Lukoschek, P. Cross, G. Torda, R. Zimmerman, B. L. Willis, The importance of coral larval recruitment for the recovery of reefs impacted by cyclone Yasi in the Central Great Barrier Reef. *PLOS ONE* **8**, e65363 (2013).

66. A. H. Baird, B. Sommer, J. S. Madin, Pole-ward range expansion of Acropora spp. along the east coast of Australia. *Coral Reefs* **31**, 1063 (2012).

67. T. Naaykens, C. C. D’Aloia, Isolation-by-distance and genetic parentage analysis provide similar larval dispersal estimates. *Mol. Ecol.* **31**, 3072–3082 (2022).

68. M. L. Pinsky, P. Saenz-Agudelo, O. C. Salles, G. R. Almany, M. Bode, M. L. Berumen, S. Andréfouët, S. R. Thorrold, G. P. Jones, S. Planes, Marine dispersal scales are congruent over evolutionary and ecological time. *Curr. Biol.* **27**, 149–154 (2017).

69. E. A. Treml, J. J. Roberts, Y. Chao, P. N. Halpin, H. P. Possingham, C. Riginos, Reproductive output and duration of the pelagic larval stage determine seascape-wide connectivity of marine populations. *Integr. Comp. Biol.* **52**, 525–537 (2012).

70. C. C. Smith, S. D. Fretwell, The optimal balance betweensSize and number of offspring. *Am. Nat.* **108**, 499–506 (1974).

71. G. A. Parker, M. Begon, Optimal egg size and clutch size: Effects of environment and maternal phenotype. *Am. Nat.* **128**, 573–592 (1986).

72. Australian Institute of Marine Science (AIMS). 2023. EcoRRAP Coral Demographic Data. https://apps.aims.gov.au/metadata/view/5d14f00f-6c24-43c1-b44e-70fe013d0757, accessed 09-Sep-2024.

73. M. C. Neel, K. McKelvey, N. Ryman, M. W. Lloyd, R. S. Bull, F. W. Allendorf, M. K. Schwartz, R. S. Waples, Estimation of effective population size in continuously distributed populations: There goes the neighborhood. *Heredity* **111**, 189–199 (2013).

74. R. S. Waples, Genetic estimates of contemporary effective population size: To what time periods do the estimates apply? *Mol. Ecol.* **14**, 3335–3352 (2005).

75. M. Nei, W.-H. Li, Linkage disequilibrium in subdivided populations. *Genetics* **75**, 213–219 (1973).

76. P. Sinnock, The Wahlund effect for the two-locus model. *Am. Nat.* **109**, 565–570 (1975).

77. M. P. Hare, L. Nunney, M. K. Schwartz, D. E. Ruzzante, M. Burford, R. S. Waples, K. Ruegg, F. Palstra, Understanding and estimating effective population size for practical application in marine species management: Applying effective population size estimates to marine species management. *Conserv. Biol.* **25**, 438–449 (2011).

78. R. Frankham, Effective population size/adult population size ratios in wildlife: A review. *Genet. Res.* **66**, 95–107 (1995).

79. G. Luikart, N. Ryman, D. A. Tallmon, M. K. Schwartz, F. W. Allendorf, Estimation of census and effective population sizes: The increasing usefulness of DNA-based approaches. *Conserv. Genet.* **11**, 355–373 (2010).

80. S. H. Clarke, E. R. Lawrence, J. Matte, B. K. Gallagher, S. J. Salisbury, S. N. Michaelides, R. Koumrouyan, D. E. Ruzzante, J. W. A. Grant, D. J. Fraser, Global assessment of effective population sizes: Consistent taxonomic differences in meeting the 50/500 rule. *Mol. Ecol.* **33**, e17353 (2024).

81. S. Wright, Evolution and the genetics of populations: A treatise in three volumes. volume 2: The theory of gene frequencies. *Q. Rev. Biol.* **45**, 393–393 (1970).

82. S. E. Sultan, H. G. Spencer, Metapopulation structure avors plasticity over local adaptation. *Am. Nat.* **160**, 271–283 (2002).

83. E. C. Johnston, Z. H. Forsman, J.-F. Flot, S. Schmidt-Roach, J. H. Pinzón, I. S. S. Knapp, R. J. Toonen, A genomic glance through the fog of plasticity and diversification in Pocillopora. *Sci. Rep.* **7**, 5991 (2017).

84. T. Capblancq, M. C. Fitzpatrick, R. A. Bay, M. Exposito-Alonso, S. R. Keller, Genomic prediction of (mal)adaptation across current and future climatic landscapes. *Annu. Rev. Ecol. Evol. Syst.* **51**, 245–269 (2020).

85. A. M. Derry, D. J. Fraser, S. P. Brady, L. Astorg, E. R. Lawrence, G. K. Martin, J. Matte, J. O. N. Dastis, A. Paccard, R. D. H. Barrett, L. J. Chapman, J. E. Lane, C. G. Ballas, M. Close, E. Crispo, Conservation through the lens of (mal)adaptation: Concepts and meta-analysis. *Evol. Appl.* **12**, 1287–1304 (2019).

86. S. N. Aitken, M. C. Whitlock, Assisted gene flow to facilitate local adaptation to climate change. *Annu. Rev. Ecol. Evol. Syst.* **44**, 367–388 (2013).

87. S. Mathur, J. M. Tomeček, L. A. Tarango-Arámbula, R. M. Perez, J. A. DeWoody, An evolutionary perspective on genetic load in small, isolated populations as informed by whole genome resequencing and forward-time simulations. *Evolution* **77**, 690–704 (2023).

88. M. B. Davis, R. G. Shaw, Range shifts and adaptive responses to quaternary climate change. *Science* **292**, 673–679 (2001).

89. T. J. Richards, K. McGuigan, J. D. Aguirre, A. Humanes, Y. Bozec, P. J. Mumby, C. Riginos, Moving beyond heritability in the search for coral adaptive potential. *Glob. Chang. Biol.* **29**, 3869–3882 (2023).

90. M. N. Dawson, Natural experiments and meta-analyses in comparative phylogeography. *J. Biogeogr.* **41**, 52–65 (2014).

91. B. Rinkevich, Y. Loya, Reproduction of the Red Sea coral Stylophora pistillata. 2. Synchronization in breeding and seasonality of planulae shedding. *Mar. Ecol. Prog. Ser.* **1**, 145–152 (1979).

92. A. Nishikawa, M. Katoh, K. Sakai, Larval settlement rates and gene flow of broadcast-spawning (*Acropora tenuis*) and planula-brooding (*Stylophora pistillata*) corals. *Mar. Ecol. Prog. Ser.* **256**, 87–97 (2003).

93. A. H. Baird, The Ecology of Coral Larvae: Settlement Patterns, Habitat Selection and the Length of the Larval Phase, thesis, James Cook University (2001).

94. A. Klueter, N. Andreakis, Assessing genetic diversity in the scleractinian coral *Stylophora pistillata* (Esper 1797) from the Central Great Barrier Reef and the Coral Sea. *Syst. Biodivers.* **11**, 67–76 (2013).

95. S. Schmidt-Roach, K. J. Miller, E. Woolsey, G. Gerlach, A. H. Baird, Broadcast spawning by Pocillopora species on the Great Barrier Reef. *PLOS ONE* **7**, e50847 (2012).

96. S. Gordon, E. Aston, M. Lechene, J. Harianto, P. Bray, W. Figueira, M. Gonzalez-Rivero, F. Legorreta, Renata. (2023). Field photogrammetry in 4D: Overview & in-field workflow Reef Restoration and Adaption Program (EcoRRAP) Standard Operational Procedure Number 14 (no. 1 of series). 10.25845/SE7T-PS86.

97. G. Pavoni, M. Corsini, F. Ponchio, A. Muntoni, C. Edwards, N. Pedersen, S. Sandin, P. Cignoni, TagLab: AI-assisted annotation for the fast and accurate semantic segmentation of coral reef orthoimages. *J. Field Robot.* **39**, 246–262 (2022).

98. C. G. B. Grupstra, M. Gómez-Corrales, J. E. Fifer, H. E. Aichelman, K. S. Meyer-Kaiser, C. Prada, S. W. Davies, Integrating cryptic diversity into coral evolution, symbiosis and conservation. *Nat. Ecol. Evol.* **8**, 622–636 (2024).

99. J. P. Hereward, T. J. Smith, R. Gloag, D. R. Brookes, G. H. Walter Tests of hybridisation in Tetragonula stingless bees using multiple genetic markers (Evolutionary Biology 2020). bioRxiv [Preprint] (2020) 10.1101/2020.03.08.982546.

100. D. A. R. Eaton, I. Overcast, ipyrad: Interactive assembly and analysis of RADseq datasets. *Bioinformatics* **36**, 2592–2594 (2020).

101. P. Danecek, A. Auton, G. Abecasis, C. A. Albers, E. Banks, M. A. DePristo, R. E. Handsaker, G. Lunter, G. T. Marth, S. T. Sherry, G. McVean, R. Durbin, 1000 Genomes Project Analysis Group, The variant call format and VCFtools. *Bioinformatics* **27**, 2156–2158 (2011).

102. S. Purcell, B. Neale, K. Todd-Brown, L. Thomas, M. A. R. Ferreira, D. Bender, J. Maller, P. Sklar, P. I. W. De Bakker, M. J. Daly, P. C. Sham, PLINK: A tool set for whole-genome association and population-based linkage analyses. *Am. J. Hum. Genet.* **81**, 559–575 (2007).

103. T. Jombart, I. Ahmed, *adegenet 1.3-1*: New tools for the analysis of genome-wide SNP data. *Bioinformatics* **27**, 3070–3071 (2011).

104. A. D. Martin, K. M. Quinn, J. H. Park, MCMCpack: Markov chain Monte Carlo in *R. J. Stat. Soft.* **42**, 1–21 (2011).

105. D. H. Alexander, K. Lange, Enhancements to the ADMIXTURE algorithm for individual ancestry estimation. *BMC Bioinformatics* **12**, 246 (2011).

106. B. Gruber, P. J. Unmack, O. F. Berry, A. Georges, DARTR: An R package to facilitate analysis of SNP data generated from reduced representation genome sequencing. *Mol. Ecol. Resour.* **18**, 691–699 (2018).

107. T. L. Schmidt, M. Jasper, A. R. Weeks, A. A. Hoffmann, Unbiased population heterozygosity estimates from genome-wide sequence data. *Methods Ecol. Evol.* **12**, 1888–1898 (2021).

108. P. Danecek, J. K. Bonfield, J. Liddle, J. Marshall, V. Ohan, M. O. Pollard, A. Whitwham, T. Keane, S. A. McCarthy, R. M. Davies, H. Li, Twelve years of SAMtools and BCFtools. *Gigascience* **10**, giab008 (2021).

109. A. T. Adamack, B. Gruber, POPGENREPORT: Simplifying basic population genetic analyses in R. *Methods Ecol. Evol.* **5**, 384–387 (2014).

110. P. G. Meirmans, The trouble with isolation by distance. *Mol. Ecol.* **21**, 2839–2846 (2012).

111. C. C. D'Aloia, S. M. Bogdanowicz, R. K. Francis, J. E. Majoris, R. G. Harrison, P. M. Buston, Patterns, causes, and consequences of marine larval dispersal. *Proc. Natl. Acad. Sci. U.S.A.* **112**, 13940–13945 (2015).

112. G. R. Almany, M. L. Berumen, S. R. Thorrold, S. Planes, G. P. Jones, Local replenishment of coral reef fish populations in a marine reserve. *Science* **316**, 742–744 (2007).

113. T. Gosselin, thierrygosselin/radiator: update, version 1.1.4, Zenodo (2020); 10.5281/ZENODO.3687060.

114. C. J. S. Sier, P. J. W. Olive, Reproduction and reproductive variability in the coral Pocillopora verrucosa from the Republic of Maldives. *Mar. Biol.* **118**, 713–722 (1994).

115. E. Pebesma, Simple features for R: Standardized support for spatial vector data. *R. J.* **10**, 439 (2018).

116. J. S. Stimson, Mode and timing of reproduction in some common hermatypic corals of Hawaii and Enewetak. *Mar. Biol.* **48**, 173–184 (1978).

117. R. S. Waples, Practical application of the linkage disequilibrium method for estimating contemporary effective population size: A review. *Mol. Ecol. Resour.* **24**, e13879 (2024).

118. R. K. Waples, W. A. Larson, R. S. Waples, Estimating contemporary effective population size in non-model species using linkage disequilibrium across thousands of loci. *Heredity* **117**, 233–240 (2016).

119. J. C. Kenyon, Models of reticulate evolution in the coral genus Acropora based on chromosome numbers: Parallels with plants. *Evolution* **51**, 756–767 (1997).
